# Compact Cas9d and HEARO enzymes for genome editing discovered from uncultivated microbes

**DOI:** 10.1038/s41467-022-35257-7

**Published:** 2022-12-15

**Authors:** Daniela S. Aliaga Goltsman, Lisa M. Alexander, Jyun-Liang Lin, Rodrigo Fregoso Ocampo, Benjamin Freeman, Rebecca C. Lamothe, Andres Perez Rivas, Morayma M. Temoche-Diaz, Shailaja Chadha, Natalie Nordenfelt, Owen P. Janson, Ian Barr, Audra E. Devoto, Gregory J. Cost, Cristina N. Butterfield, Brian C. Thomas, Christopher T. Brown

**Affiliations:** Metagenomi Inc. 1545 Park Ave, Emeryville, CA 94608 USA

**Keywords:** Metagenomics, DNA restriction-modification enzymes

## Abstract

Programmable, RNA-guided nucleases are diverse enzymes that have been repurposed for biotechnological applications. However, to further expand the therapeutic application of these tools there is a need for targetable systems that are small enough to be delivered efficiently. Here, we mined an extensive genome-resolved metagenomics database and identified families of uncharacterized RNA-guided, compact nucleases (between 450 and 1,050 aa). We report that Cas9d, a new CRISPR type II subtype, contains Zinc-finger motifs and high arginine content, features that we also found in nucleases related to HEARO effectors. These enzymes exhibit diverse biochemical characteristics and are broadly targetable. We show that natural Cas9d enzymes are capable of genome editing in mammalian cells with >90% efficiency, and further engineered nickase variants into the smallest base editors active in *E. coli* and human cells. Their small size, broad targeting potential, and translatability suggest that Cas9d and HEARO systems will enable a variety of genome editing applications.

## Abstract (web summary)

Programmable, RNA-guided nucleases are diverse enzymes that have been repurposed for biotechnological applications. Here, the authors mine an extensive genome-resolved metagenomics database and identified uncharacterized families of RNA-guided, compact nucleases.

## Introduction

RNA-guided nucleases employ a variety of mechanisms for guide acquisition, targeting, and DNA cleavage (reviewed in ref. 1). Among these, CRISPR systems are involved in microbial defense against viral infections^2^ and are extensively used as genome editing tools. The most well-studied of these enzymes, SpCas9, has been developed for biotechnological applications, and has been used clinically to treat genetic diseases such as the blood disorders sickle cell disease and B-thalassemia^3,4^, as well as the ocular disease Leber’s congenital amaurosis (reviewed in ref. 5). In addition to editing via dsDNA breaks, these programmable systems have been engineered to modify select nucleotides in the genome through a process called base editing^6,7^. These systems typically consist of an engineered Cas9 effector that cleaves only one strand of DNA (i.e., a nickase), which is fused to either cytosine or an adenosine deaminase to achieve C to T (CBE) or A to G (ABE) conversions, respectively. Yet, current Cas9-based nuclease and base editor systems pose a challenge for therapeutic delivery due to their large size^8^.

Proteins containing both RuvC and HNH catalytic domains are limited to Cas9 and previously identified IscB proteins^9^. Many Cas9 orthologs with diverse characteristics have been described. For example, a small, 984 aa Cas9 ortholog with nuclease activity in mouse and human cells was identified in *Campylobacter jejuni*^10^, a small SlugCas9 with a simple NGG PAM was engineered for both high specificity and activity^11^, and more recently, PpCas9, a 1055 aa Cas9 ortholog with an NRT PAM has been shown to be active in human cells^12^. However, comparatively little is known about smaller and more divergent homologs that have only recently been demonstrated to be active RNA-guided nucleases, including IscB and TnpB^9,13,14^. IscB (insertion sequences Cas9-like) were initially reported to be associated with insertion sequences IS200/IS605^9^. Recently, Altae-Tran, Kannan, and colleagues reported that IscB are RNA guided and programmable, established an evolutionarily link between IscB and Cas9, and showed these systems can be used for genome editing in mammalian cells, albeit with very low editing efficiencies^13^.

Given the potential of RNA-guided systems for genome editing and other applications, we sought to identify and biochemically characterize representatives of nuclease families distantly related to previously described Cas9 and IscB effectors. We mined billions of proteins predicted from microbial genomic fragments assembled from metagenomic sequence data and discovered divergent IscB and Cas9 nuclease homologs. Unlike most biochemically characterized Cas9 proteins, several of these families contain representatives from the archaeal domain. We report on CRISPR Class 2 type II-D and II-C2 nucleases, as well as compact nucleases related to IscB^9,13^ and HEARO systems^15^. For these systems, we demonstrate efficient activity in vitro and in cells, and further engineered nickases into the smallest adenine and cytosine base editing systems to date that have demonstrated activity in bacterial and mammalian cells. Owing to the shared but unique biochemical attributes of these systems, we refer to them as SMART (SMall Arginine-Rich sysTems).

## Results

### Small effectors containing RuvC and HNH domains

**Fig. 1:**
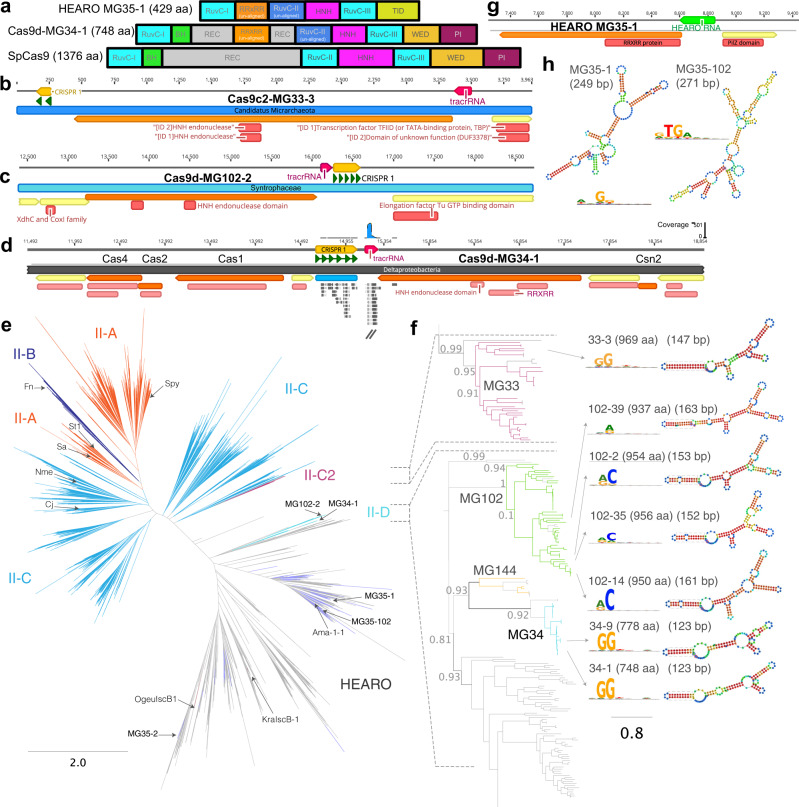
Cas9d and HEARO enzymes are dsDNA nucleases with diverse targeting ability. **a** Predicted domain architecture of Cas9d and HEARO nucleases recovered here vs. SpCas9 (not to scale). **b**–**d** Genomic context of the CRISPR-associated systems Cas9c2-MG33-33 (**b**), Cas9d-MG102-2 (**c**), and Cas9d-MG34-1 (**d**). The tracrRNA and CRISPR array orientations were confirmed by in vitro cleavage activity with the effector. Adaptation module genes (Cas1, Cas2, Cas4, and putative Csn2) were identified in a few cases. Environmental RNASeq reads in (**d**) mapped in the forward orientation to the array and intergenic region encoding a tracrRNA. Genes not associated with the described nucleases are represented by yellow arrows. **e** Phylogenetic protein tree of Cas9d, Cas9c2, and HEARO nucleases vs. reference sequences. The tree was inferred from a multiple sequence alignment of the shared RuvC-II/HNH/RuvC-III domains across >11,800 sequences. The Cas9c2-MG33 family of nucleases (burgundy branches) clusters with two archaeal Cas9 references, while other CRISPR-associated Cas9d (teal branches) cluster with sequences recently classified as type II-D. HEARO nucleases (lilac branches) cluster with HEARO ORF and IscB sequences (gray branches). Reference Cas9 sequences correspond to: Cj, CjCas9; Nme, NmeCas9; Sa, SaCas9; St1, St1Cas9; Fn, FnCas9; Spy, SpCas9. Reference HEARO (Ama-1-1) and IscB (KraIscB-1 and OgeuIscB1) effectors are also shown. The distance between tips was estimated as two substitutions per site (horizontal bar). **f** Phylogenetic clades of type II-C2 and II-D families. The clades are a zoom-in representation of the phylogenetic tree on 1E. Local support values for internal family split nodes are shown and range from 0 to 1. SeqLogo representation of consensus target motif sequences and sgRNA designs from biochemical cleavage activity assays for active Cas9c2 and Cas9d nucleases are shown. The distance between tips is estimated as 0.8 substitutions per site (horizontal bar). **g** Genomic context of the HEARO nuclease MG35-1 with its encoded RNA. **h** HEARO RNA secondary structure for two active nucleases. SeqLogo representation of consensus target motif sequences are shown.

Analysis of tens of thousands of high-quality metagenomics assemblies uncovered diverse proteins containing both RuvC and HNH nuclease domains, including several proteins notable for their unusually small size (<900 aa) (Fig. 1a, Supplementary Data File 1, and Supplementary Fig. 1A). Initial homology searches to known databases revealed no close hits but did uncover distant similarity to proteins of unknown function, HNH endonucleases, and archaeal Cas9 CRISPR effectors (<40% amino acid identity; Supplementary Fig. 1B). The effectors contain six putative HNH and RuvC catalytic residues when aligned with the SaCas9 reference sequence, although the RuvC-I, bridge helix, and recognition domains align poorly (Supplementary Figs. 2, 3). The presence of these domains was further validated with 3D structure prediction conducted from alignments to the SaCas9 crystal structure (Supplementary Fig. 4). Most proteins contain hits to the Pfam domain PF14239 (RRXRR protein), which is associated with diverse endonuclease activities.

Along with sequences previously classified as Type II-C2, which encompasses only two Archaeal Cas9 sequences reported to date (reviewed in refs. 16, 17), these proteins contain unusually high arginine and lysine amino acid content relative to the average content reported earlier for protein sequences from the Uniref50 database^18^, and likely contribute to an elevated charge and isoelectric point (Supplementary Data File 2). On average, the percent arginine and lysine composition deviates from a linear trend observed for other residues in these enzymes and from the residue composition of proteins in the Uniref50 database (Supplementary Fig. 5A). In addition, their methionine content is statistically lower than the content observed in proteins from the Uniref50 database (Supplementary Fig. 5B). The enzymes contain significantly more RRXRR motifs and zinc-binding ribbon motifs (CX(2-4)C, CX(2-4)H, or HX(2-4)C) than previously described Cas9 effectors of the II-A, II-B, or II-C subtypes (Supplementary Fig. 6). For example, a pair of Zn-binding ribbon motifs delineate a Zn-finger (CX(2,4)CX(27,31)CX(2,4)[C/H]) within the HNH domain, likely involved in target recognition and catalysis (Supplementary Figs. 2, 3). Together these results indicate that these compact RuvC and HNH domain-containing enzymes are likely dsDNA nucleases, but with unique biochemical properties.

In order to best understand the evolutionary relationships between these sequences, a multiple sequence alignment of full-length effectors was built from these newly reported effector protein sequences along with those previously classified as type II effectors^17,19^, and >10,300 recently reported Cas9 homologs and IscB sequences^13^. After trimming, a well-aligned region encompassing the RuvC-II/HNH/RuvC-III domains was retained for phylogenetic analysis (see Methods). These analyses identified divergent clades of effectors clustering away from known Cas9 sequences currently classified as II-A, II-B, and II-C (Fig. 1e). Two clades found phylogenetically closer to classified type II effectors were more likely to be encoded adjacent to CRISPR arrays (Fig. 1b–e and Supplementary Data File 1). The MG33 family of nucleases clusters with type II-C2 sequences and greatly expands this clade (Fig. 1b, e, f, mauve branches). This family contains representatives between 900 and 1050 aa in length, a length distribution that overlaps with the smallest classified type II-C references (Supplementary Fig. 1A). A more distant clade (Fig. 1c–f, teal, green and yellow branches) contains “early Cas9” sequences recently classified as type II-D^13^ (Fig. 1e, f, light gray branches). Therefore, we refer to the CRISPR-associated nucleases recovered here as Cas9c2 and Cas9d, respectively.

### Cas9d and Cas9c2 effectors are active, RNA-guided dsDNA CRISPR endonucleases

Cas9d and Cas9c2 effectors range between approximately 600 aa and 1050 aa (Supplementary Data File 1 and Supplementary Fig. 1A). Except for the recognition domain, the domain architecture resembles that of Cas9, although effectors share low sequence similarity with reference Cas9 sequences (Supplementary Figs. 1B, 2). Unlike previously described Cas9, Cas9d and Cas9c2 enzymes contain Zn-binding ribbon motifs in the recognition domain and in the vicinity of the RuvC-II domain. In all cases, the proteins are encoded adjacent to CRISPR arrays and predicted tracrRNAs, but only in a few cases are they found in the same operon with adaptation genes csn2, cas1, cas2, and/or cas4 (Fig. 1d and Supplementary Figs. 7A,  8B). Environmental expression data confirmed in situ transcription of the CRISPR array and tracrRNA (Fig. 1d and Supplementary Fig. 7A), and we identified a phage genome being targeted by one of the spacers encoded in one Cas9d CRISPR array (Supplementary Fig. 7B). Therefore, these CRISPR systems are active in their natural environments, likely as RNA-guided nucleases involved in phage defense.

**Fig. 2:**
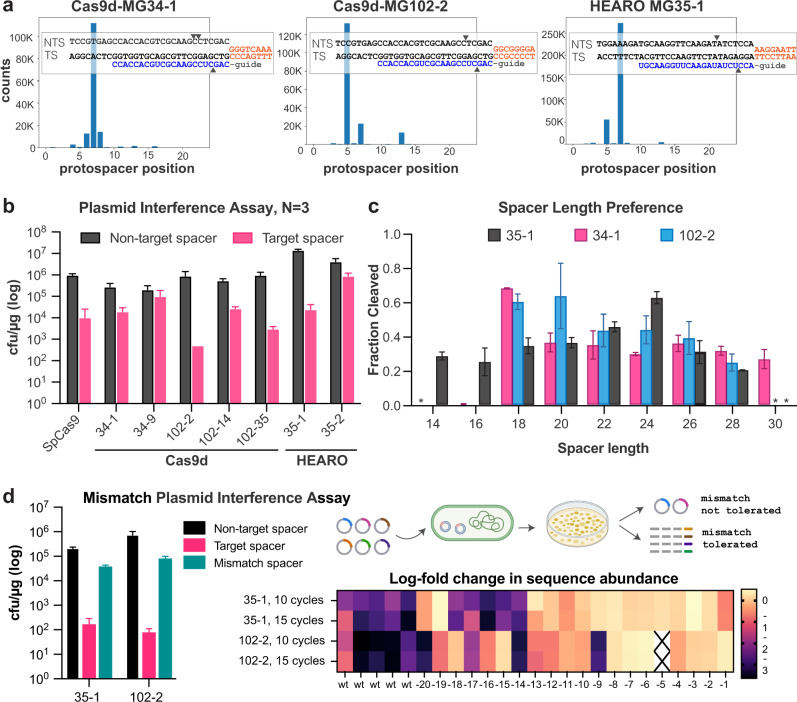
Cas9d and HEARO enzymes are dsDNA nucleases. **a** Histograms of cleavage position preference for three active nucleases on the non-target strand from NGS. The insets show a staggered cut, where cleavage at position 3 occurs on the target strand (TS), while cleavage at positions 5–7 from the PAM occurs on the non-target strand (NTS). The TS cleavage site was determined via Sanger run-off sequencing. **b** Bar plot of colony forming unit (cfu) measurements (in log-scale) showing *E. coli* growth repression in the target condition vs. the non-target controls. Plasmid interference assays for each nuclease were done in triplicate along with the SpCas9 positive control. Whiskers represent the standard deviation from the mean. **c** Measurement of in vitro DNA cleavage efficiency with varying spacer lengths indicates a preference for 18–20 bp spacers for Cas9d nucleases, while the HEARO nuclease MG35-1 prefers 24 bp spacers. (*) spacer lengths 14 bp (for Cas9d-MG34-1), and 30 bp (for HEARO MG35-1 and Cas9d-MG102-2) were not evaluated. Whiskers represent the standard deviation from the mean. **d** Mismatch plasmid interference assays indicate high specificity for target spacers at positions −1 to −13 from the PAM. Left: Bar plot of colony forming unit (cfu) measurements (log-scale) showing *E. coli* growth repression in the target condition vs. a spacer containing mismatches, as well as the non-target controls. Top right: Diagram of the mismatch plasmid interference assay. *E. coli* containing two plasmids for nuclease expression and guide expression are transformed with a library of target plasmids with mismatches in the protospacer. Mismatches were assayed for one to three alternate base pairs at each position. Bottom right: heatmap showing mismatch tolerance at each position of the target spacer. For the target spacer and spacers with tolerated mismatches, growth is expected to be repressed (purple). Positions with required base pairing will not cut efficiently and will be relatively enriched in the output library (yellow). Plasmid interference (kill) assays with the library for each nuclease were done in duplicate. Whiskers represent the standard deviation from the mean.

Putative single guide RNAs (sgRNA) were engineered using the environmental RNA expression data or from CRISPR repeat and tracrRNA predictions (Supplementary Data File 3), and then tested in vitro in PAM enrichment assays (described in materials and methods). Assays confirmed dsDNA cleavage for 16 Cas9d and Cas9c2 nucleases with various sgRNA designs (Figs. 1f,  2 and Supplementary Fig. 9A), whose structures differ from known Cas9 sgRNAs^20,21^ (Supplementary Fig. 9B). Several of the nucleases require a 3′ NGG PAM, while other representatives require a 3′ NRC or NAR PAMs for target recognition and cleavage. Near complete genome bins encoding three of the active Cas9d nucleases were recovered from unclassified *Deltaproteobacteria*, including two from the *Syntrophaceae* family (Cas9d-MG102-2 and Cas9d-MG102-14), while one partial genome belongs to an unclassified Class of the Candidatus *Micrarchaeota* Phylum (Cas9c2-MG33-3) (Supplementary Data File 4). When evaluating which other CRISPR systems are encoded in these genomes, the described type II-C2 and type II-D systems are generally the only CRISPR systems present (Supplementary Data File 4).

Sequencing the cleavage products of the Cas9d-MG34-1 and Cas9d-MG102-2 nucleases shows that these enzymes create a staggered double-strand DNA break (Fig. 2a). Analysis of the cleavage sites indicates preferential cleavage of five to seven nucleotides from the PAM (Fig. 2a and Supplementary Data File 3). These results suggest a rarely observed biochemical cleavage mechanism compared with most Cas9 enzymes, which create blunt ends or staggered cleavage that are preferentially at positions three to five from the PAM^19,22^.In vitro cleavage assays with in vitro transcription/translation reactions and with purified protein indicate that Cas9d-MG34-1 and Cas9d-MG102-2 are most efficient in vitro with 18 and 20 nucleotide spacers (Fig. 2c). Furthermore, activity was confirmed using *E. coli* plasmid interference assays, showing tenfold (Cas9d-MG34-1) to over 500-fold (Cas9d-MG102-2) growth repression for five Cas9d nucleases with the specified targeting spacer (Fig. 2b and Supplementary Fig. 10).

To determine the specificity of Cas9d enzymes, an interference assay was developed to measure differences in the efficiency of targeting plasmids containing kanamycin resistance genes with perfectly matched and mismatched protospacers (Fig. 2d). If a mismatch is tolerated, the enzyme is expected to cleave the antibiotic resistance gene and growth impairment will be observed. The Cas9d-MG102-2 nuclease does not tolerate mismatches along the first 13 positions from the PAM (except for position nine), while variable mismatch tolerance was observed in distal positions 14, 17, and 20 from the PAM (Fig. 2d and Supplementary Fig. 11A). Results suggest that the long “seed” region of the spacer enables Cas9d nucleases to be highly specific. In addition, Cas9d do not exhibit collateral ssDNA cleavage in vitro (Supplementary Fig. 12). To the best of our knowledge, our results report the first active representatives of the type II-D subclass, the first active type II endonuclease from the archaeal domain and highlight these recently discovered natural nucleases.

### Genome editing in human cells with Cas9d-MG102-2

**Fig. 3:**
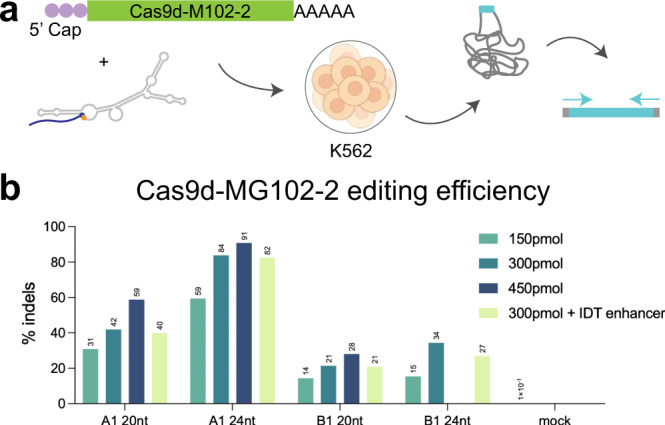
Cas9d-MG102-2 is a highly active nuclease in human cells. **a** Flow diagram of Cas9d-MG102-2 mRNA delivery to K562 cells. The sgRNA with a 20 or 24 bp guide and the human codon-optimized nuclease mRNA were nucleofected into cells, genomic DNA was extracted and editing at the expected target site was determined via amplicon NGS. **b** Nuclease activity was evaluated for the Cas9d-MG102-2 nuclease at two targeting sites in the TRAC locus (guides A1 and B1) with increasing concentrations of sgRNA (150, 300, and 450 pmol/reaction). The mock control represents background editing levels at the target region in the absence of mRNA and guide.

We delivered Cas9d nucleases via mRNA to human cells targeting the T cell receptor alpha constant locus (TRAC) and demonstrated over 90% editing activity at one of two target sites with the Cas9d-MG102-2 nuclease (Fig. 3). As observed in in vitro experiments (Supplementary Fig. 13), increasing the amount of sgRNA greatly improved editing efficiency at both target loci (Fig. 3). Although we were able to confirm localization of the Cas9d-MG34-1 system to the nucleus of human cells (fused with nuclear localization signals, NLS; Supplementary Fig. 14), the proteins form foci when delivered in the absence of their guide RNA, possibly undergoing liquid-phase separation. We were unable to detect nuclease-induced InDel formation for this nuclease. However, we anticipate that further protein and guide optimization for these and other Cas9d and Cas9c2 nucleases will improve the efficiency of these systems for genome editing and other biotechnological applications.

### HEARO effectors are compact, arginine-rich, RNA-guided endonucleases

Phylogenetic analysis indicated that nucleases of less than 600 aa in length (Fig. 1e, lilac branches) cluster together forming two main clades with previously described HEARO (“HNH Endonuclease-Associated RNA and ORF”)^15^ and IscB (“insertion sequences Cas9-like”)^9,13^ protein sequences (Fig. 1e, dark gray branches and Supplementary Fig. 8A). HEARO systems were first described in bioinformatics analysis of highly structured RNAs^15^, but the function of the associated HEARO HNH endonuclease ORF remained unknown^23^. Here we confirmed that HEARO ORFs contain RuvC and HNH catalytic domains, high arginine and lysine content, and numerous Zn-binding ribbon motifs (Supplementary Figs. 3, 8A). Based on phylogenetic analysis, HEARO ORFs belong to the same family of nucleases later described as IscB. Kapitonov and colleagues reported IscB (HEARO) effectors having homology with Cas9 nucleases based on the presence of RuvC and HNH domains^9^, and a PLMP domain was subsequently described in this same group of enzymes^13^. We used de novo 3D structure prediction to show that these proteins contain an arginine-rich region, usually containing an RRXRR motif (Supplementary Figs. 3, 4). The arginine-rich region was suggested to be analogous to the bridge helix in Cas9^9^; however, structure prediction indicated that neither this region nor the RuvC-I domain aligns well in 3D space with the bridge helix and RuvC-I domains of the reference SaCas9 3D structure (Supplementary Fig. 4). HEARO enzymes lack a PAM interacting domain. Instead, a recently reported C-terminal TAM interacting domain^24^ containing Zn-binding ribbon motifs is involved in target motif recognition (Supplementary Figs. 3,  4). Only a few HEARO nucleases were found associated with TnpA transposases of the insertion sequences IS200/IS605 (Supplementary Fig. 15A), unlike early associations reported by Kapitonov and colleagues^9^. Furthermore, HEARO sequences were only sparsely found in the vicinity of IS200/IS605 TnpA transposases by Altae-Tran, Kannan, and colleagues^13^.

Searches for non-coding RNAs (ncRNA) associated with HEARO nucleases found that 65% of 5′ untranslated regions (UTRs) contain hits to HEARO RNAs from the RFam database (RF02033) (Supplementary Data File 1). Recently, Altae-Tran, Kannan, and colleagues reported that the 5′ UTR of IscB (HEARO) encodes a single guide RNA required for dsDNA nuclease activity, which the authors refer to as Omega RNA^13^. In confirmation of the requirement of a guide RNA for function, we observed in situ natural expression of the 5′ UTR of HEARO systems (Supplementary Fig. 16A), which was recapitulated by in vitro transcription assays (Supplementary Fig. 16B). HEARO RNAs reported here are highly conserved structurally when compared to HEARO RNAs reported bioinformatically by Weinberg and colleagues^15^, and with Omega RNA structures from the Altae-Tran, Kannan and colleagues’ report^13^ (Supplementary Fig. 16D, E). Therefore, in recognition of the features that unite IscB and HEARO systems (broad taxonomic origin, enrichment of arginine residues, clustering in the phylogenetic tree), as well as of the chronological discovery of the highly structured guide RNAs associated with these enzymes, we advocate for the classification of these systems as HEARO (Fig. 1e).

### HEARO clades contain virus-associated systems

Although protein domains, catalytic residues, and 3D models suggest an evolutionary relationship with Cas9, the HEARO systems are not CRISPR-associated (Supplementary Data File 1) (see also ref. 13). They are widely distributed in bacterial and archaeal genomes (Supplementary Fig. 1A and Supplementary Data File 1) and over 16% of genomic fragments encoding these effectors were classified as likely viral or prophage-derived (Supplementary Fig. 15B, C and Supplementary Data File 1), implicating viruses in the evolution of these systems.

### HEARO nucleases are highly active and specific in *E. coli*

We evaluated HEARO nuclease cleavage activity in vitro and identified required targeting motifs by reprogramming the 5’ “spacer” region of their HEARO RNA (Fig. 1g, h, Supplementary Figs. 16A–C, 17, and Supplementary Data File 2), as described by Altae-Tran, Kannan and colleagues^13^. Moreover, plasmid interference assays in *E. coli* show that HEARO nucleases are highly active compared to SpCas9 (>570-fold repression for MG35-1 vs. ~98-fold repression for SpCas9, Fig. 2b and Supplementary Fig. 10A, D) and specificity experiments indicate low tolerance for mismatches in the target sequence up through position 13 (Fig. 2d and Supplementary Fig. 11B).

### Base editing with engineered Cas9d and HEARO effectors

**Fig. 4:**
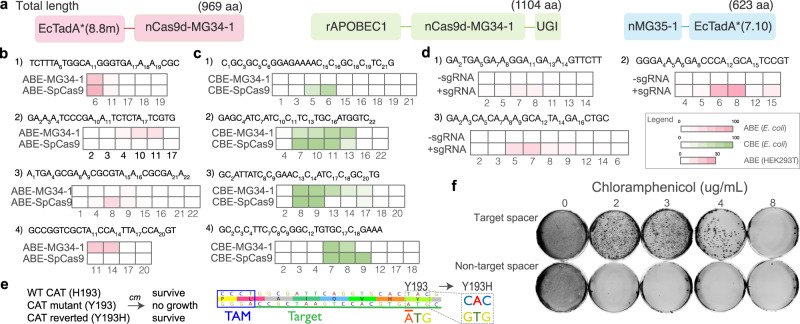
Base editing with engineered Cas9d-MG34-1 and HEARO MG35-1 nickases. **a** Diagrams of base editor constructs with total amino acid length (not to scale). **b**–**d** Base editing at multiple genomic target loci. Base editing in *E. coli* with ABE-MG34-1 (**b**) and CBE-MG34-1 (**c**) vs. reference SpCas9 base editors at four target loci. **d** Base editing in human HEK293T cells with an ABE-MG34-1 (nickase fused with TadA*(8.8 m) deaminase) at three target loci. The target sequence for each locus in **b**–**d** is shown above each heatmap. Expected edit positions are represented on the sequence by a subscript number and at each position on the heatmap (squares). Heatmaps in **b**–**d** represent the percentage of NGS reads supporting an edit at each position. Values in **b** and **c** represent the mean of two independent experiments, while values in **d** represent the mean of three independent biological replicates. **e** *E. coli* survival assay. *E. coli* was transformed with a plasmid containing the nMG35-1-ABE, a nonfunctional chloramphenicol acetyltransferase (CAT H193Y) gene, and a sgRNA that either targets the CAT gene (target spacer) or not (non-target spacer). Left: *E. coli* survival under chloramphenicol selection is dependent on the ABE base editing the nonfunctional CAT gene to its wild-type sequence. Right: Diagram showing the target sequence with the nuclease’s required TAM. The “A” base at position 17 from the TAM is expected to edit to “G” to revert the tyrosine residue to histidine and restore chloramphenicol (cm) resistance. **f** Transformed *E. coli* was grown on plates containing chloramphenicol concentrations of 0, 2, 3, 4, and 8 ug/mL. Plates also contain 100 ug/mL Carbecillin and 0.1 mM IPTG. Experiments were performed in duplicate.

Given the small size of the Cas9d nucleases and the possibility of converting them into nickases, we sought to determine if they could be engineered into compact systems for base editing. We created a Cas9d-MG34-1 mutant (D10A) for functional disruption of nuclease activity of the RuvC-I domain. The D10A variant is predicted to be a nickase based on alignments with the SaCas9 reference sequence (Supplementary Fig. 2). Nickase activity has been demonstrated for a nSaCas9 mutant with the catalytic residue Aspartate at position 10 (D10) changed to Alanine^25^. We fused the engineered variant to TadA*(8.17 m) adenosine deaminase^26^ for adenine base editor (ABE) development (ABE-MG34-1), and to both rAPOBEC1 cytosine deaminase and PBS1 uracil glycosylase inhibitor (UGI) from BE3^27^ for cytosine base editor (CBE) development (CBE-MG34-1) (Fig. 4a and Table 1). Both ABE-MG34-1 and CBE-MG34-1 edited target loci in the *E. coli* genome at levels and within editing windows comparable to reference SpCas9 base editors (Fig. 4b, c). However, ABE-MG34-1 was capable of editing at target loci not edited by SpCas9 BE (e.g., Fig. 4b, target sites 2 and 4), and vice versa. Furthermore, an ABE-MG34-1 system fused with TadA(8.8 m) deaminase^26^ is capable of base editing in human cells with up to 22% editing efficiency across three different genomic targets (Fig. 4d). Our results demonstrate that Cas9d nucleases can be translated for human base editing applications with therapeutic value.

**Table 1:** Nucleases and base editor amino acid length vs. reference SpCas9

Enzyme	Length (aa)	ABE length (aa)	CBE length (aa)
MG34-1	748	969	1104
MG35-1	429	623	–
SpCas9	1376	1588	1723
CasMINI (type V)	529	–	–

Base editor (ABE or CBE) size for constructs tested here comprise the effector, linkers, NLS, and one deaminase monomer.

To determine whether the HEARO enzymes can also be used as base editors, we constructed an ABE by fusing a TadA*(7.10) adenosine deaminase monomer^26^ to the C-terminus of an engineered putative nickase MG35-1 containing a D59A mutation (Fig. 4a, Table 1, and Supplementary Fig. 3). The A to G editing of this ABE was tested in a positive selection single-plasmid *E. coli* system in which the ABE is required to revert a chloramphenicol resistance mutation to survive chloramphenicol selection (Fig. 4e). The single plasmid contains a sgRNA with a spacer either targeting the mutant chloramphenicol acetyltransferase (CAT) gene or a scrambled, non-targeting spacer (control). *E. coli* transformed with the ABE-MG35-1 base editor successfully edited the CAT gene as shown by colonies growing on plates containing 2, 3, and 4 ug/mL of chloramphenicol (Fig. 4f and Supplementary Fig. 18). Sanger sequencing confirmed that 26 of 30 colonies from three plates contained the expected Y193H reversion (Supplementary Table 1 and Supplementary Fig. 19). No colonies were seen on any of the chloramphenicol-containing plates for *E. coli* cells transformed with the non-targeting spacer. While the 0 ug/mL condition was used as a transformation control, we found that one of the ten colonies picked from this plate contained the Y193H reversion for the target condition, indicating a detectable level of editing without chloramphenicol selection. Results indicate that the HEARO MG35-1 engineered nickase is a successful component for base editing. At 623 aa long, the ABE-MG35-1 represents the smallest, nickase-based adenine base editor to date.

## Discussion

Although CRISPR Cas9 nucleases have been extensively developed for genome editing, their large size complicates delivery for many applications. Here, we describe active members from new and recently discovered classes of programmable, RNA-guided compact nucleases (400 to ~1000 aa). While phylogenetic analysis shows that Cas9d and Cas9c2 systems are more closely related to known Cas9 than HEARO nucleases, it is notable that both classes of nucleases share unique, newly described protein features. These enzymes are enriched in arginine and lysine content, encode a Zn-finger within the HNH domain, and contain multiple Zn-binding ribbon motifs in their recognition domains, while known Cas9 sequences lack these motifs. The high arginine and Zn-binding ribbon motif content, along with the observation that Cas9d-MG34-1 may undergo liquid-phase separation, suggest that Cas9d nucleases may contain intrinsically disordered regions, which we predict add flexibility for interacting with large guide RNAs and target DNA. Intrinsically disordered regions are segments of proteins that lack a stable tertiary structure in their native, unbound state^28^, are known to be enriched in positively charged arginine residues that interact with polyanions (such as RNA)^29^, and are often found as linkers between Zn-binding ribbons to help with “search function”^30^, all of which are features found in Cas9d and HEARO nucleases. We, therefore, refer to these systems collectively as SMART (SMall Arginine-Rich sysTems).

Much of the smaller size of HEARO systems is due to their compact REC domains, a feature that we hypothesized is linked to the larger guide RNAs required by these enzymes. Indeed, Shuler and colleagues recently demonstrated through a cryo-EM structure that the Omega RNA of an IscB (HEARO) nuclease plays the role of the missing REC domain^24^. The discovery of HEARO nucleases in viral genomes represents untapped reservoirs of these enzymes, and, thus, additional opportunities to identify active systems for genome editing. Cas9d and Cas9c2 effectors are diverse families with multiple active enzymes and short (permissive) PAM sequences. This contrasts with other type II nucleases where smaller enzymes typically have larger (more restrictive) PAMs (for example, see ref. 19). We anticipate that Cas9d and HEARO systems will have major advantages for base editing applications, due to their small size. Although small CRISPR type V effectors have been used for base editing^31^, they have restricted applications because, unlike the SMARTs, the effectors require dimerization and cannot be converted to nickases.

It is noteworthy that the efficiency and specificity results reported here are for natural enzymes with no optimization (for example, of guides or protein sequences). Previous studies of uncharacterized CRISPR systems reported much lower initial (unoptimized) measurements of genome editing potential^11,12,32^. Additional optimization for efficiency and specificity will likely improve enzyme activities; however, the initial high levels of activity and specificity is a promising starting point that is indicative of their translatability. Overall, our results describe the most compact nucleases with demonstrated activity in human cells and highlight the potential for translating these systems for therapeutic applications, including for in vivo genome editing where delivery is largely constrained by system size.

## Methods

### Sample collection and sequence data processing

Seven animal microbiome (stool) samples from abandoned specimens from unknown animals were collected. Collection proceeded without disturbing any animals, and therefore, ethical approval for the use of animal specimens was not required. Additionally, high-temperature biofilm and sediment samples were collected and stored on ice or in Zymo DNA/RNA Shield after collection. DNA was extracted from samples using either the Qiagen DNeasy PowerSoil Kit or the ZymoBIOMICS DNA Miniprep Kit. DNA sequencing libraries were constructed (Illumina TruSeq) and sequenced on an Illumina HiSeq 4000 or Novaseq at the Vincent J. Coates Genomics Sequencing Laboratory at UC Berkeley, with paired 150 bp reads with a 400–800 bp target insert size. Publicly available metagenomic sequencing data from diverse environments were downloaded from the NCBI SRA. Sequencing reads were trimmed using BBMap (Bushnell B., sourceforge.net/projects/bbmap/) and assembled with Megahit^33^.

### Bioinformatic analyses

Protein sequences were predicted with Prodigal^34^. HMM profiles of known type II CRISPR nucleases were built and searched against all predicted proteins using HMMER3 (hmmer.org). Predicted proteins were annotated by searching Pfam^35,36^ (http://pfam.xfam.org/) HMMs using HMMER3. Putative ncRNA annotations were identified by searching Rfam^37,38^ (http://rfam.xfam.org/) with Infernal^39^ (http://eddylab.org/infernal/). CRISPR arrays were predicted on assembled contigs with Minced (https://github.com/ctSkennerton/minced). Taxonomy was assigned to proteins with Kaiju^40^ and contig taxonomy was determined by finding the consensus of all encoded proteins.

Although the convention is to name CRISPR nucleases based on the organism that encodes them, it is not possible to do so accurately in cases when the strains have not yet been characterized. Therefore, to best adhere to the convention, we have named these proteins with the suffix MGX-Y, where MG indicates that the proteins are derived from metagenomic fragments, X represents the family identifier and Y indicates the member identifier. For example, Cas9d-MG34-1, a Cas9d enzyme recovered from metagenomics data, is the first member of family 34.

Predicted and reference type II effector proteins (e.g. SpCas9, SaCas9, and AsCas9), as well as Cas1 proteins and ribosomal proteins were aligned with MAFFT^41,42^ with parameters G-INSI, and phylogenetic trees were inferred using FastTree2^43^. For the phylogenetic tree in Fig. 1e, over 12,000 sequences comprising enzymes reported here, as well as IscB/IsrB^13^, HEARO ORF^15^, and previously classified Cas9 sequences^19^ were aligned with MAFFT with parameters --globalpair --large. Because the RuvC-I, bridge helix, REC, WED, and PI domains aligned poorly, the alignment was trimmed to retain the region comprising the RuvC-II, HNH, and RuvC-III domains, as given by the coordinates of SaCas9^44^, and the trimmed sequences were realigned with MAFFT with parameters --globalpair --large. The final phylogenetic tree was then inferred from this final alignment with FastTree2.

Binning of microbial genomes was initially done automatically with MetaBAT^45^. Genomic bins were refined with Anvi’o^46^ using GC content, mean coverage, and taxonomic information. The number of CRISPR loci was estimated based on the presence of CRISPR arrays predicted by Minced (https://github.com/ctSkennerton/minced) and Cas gene annotations surrounding the arrays. The taxonomic assignment of genome bins was adapted from ref. 47. Briefly, a set of 13 ribosomal proteins (L2, L3, L4, L5, L6, L14, L18, L22, L24, S3, S8, S17, and S19) were extracted from curated genome bins, their individual alignments were concatenated, and a FastTree2 phylogenetic tree was constructed from the concatenated alignment in Geneious Prime 2021 (https://www.geneious.com). Structure prediction for Cas9d and HEARO nucleases was done with Novafold versions 16 and 17, and models were visualized with Protean3D (licensed from DNASTAR, www.dnastar.com). Models were aligned with the cryo-EM structure of SaCas9 downloaded from RPDB (https://www.rcsb.org/structure/5AXW).

### PAM determination

Putative sgRNAs were identified from RNAseq reads mapped to contigs containing target effectors. The tracrRNA-containing region from RNAseq data was folded with the repeat sequence from the CRISPR array in Geneious Prime 2021 with the Vienna RNAfold tool and the “RNA (Andronescou 2007)” energy model, and the resulting helix was trimmed and concatenated with a GAAA tetra-loop. Multiple lengths of repeat-anti-repeat helix trimming were tested, as well as different spacer lengths and different tracrRNA termination points. The sgRNA was constructed via assembly PCR and purified with SPRI beads or ordered as a gene fragment (IDT), and then in vitro transcribed (IVT, HiScribe T7 kit, New England Biolabs) following the manufacturer’s recommended protocol for short RNA transcripts. RNA reactions were cleaned with the Monarch RNA kit and checked for purity via a Tapestation (Agilent).

Cleavage and PAM determination assays were performed with PURExpress (New England Biolabs). Briefly, the protein was codon-optimized for *E. coli* and cloned into a vector with a T7 promoter and C-terminal His tag. The gene was PCR amplified with primer binding sites 150 bp upstream and downstream from the T7 promoter and terminator sequences, respectively. This PCR product was added to PURExpress (New England Biolabs) at 5 nM final concentration and expressed for 2 h at 37 ˚C. A cleavage reaction was assembled in 10 mM Tris pH 7.5, 100 mM NaCl, and 10 mM MgCl_2_ with a five-fold dilution of PURExpress, 5 nM of an 8 N PAM plasmid library, and 50 nM of sgRNA targeting the PAM library.

The cleavage products from the PURExpress reactions were recovered via clean-up with SPRI beads (AMPure Beckman Coulter or HighPrep Sigma-Aldritch). The DNA was blunted via the addition of Klenow fragments and dNTPs (New England Biolabs). Blunt-end products were ligated with a 100-fold excess of double-stranded adapter sequences and used as template for the preparation of an NGS library, from which PAM requirements were determined from sequence analysis.

Raw NGS reads were filtered by Phred quality score >20. The 14–24 bp representing the known DNA sequence from the backbone adjacent to the PAM was used as a reference to find the PAM-proximal region and the 8 bp adjacent were identified as the putative PAM. The distance between the PAM and the ligated adapter was also measured for each read. Reads that did not have an exact match to the reference sequence or adapter sequence were excluded. PAM sequences were filtered by cut site frequency such that only PAMs with the most frequent cut site ±2 bp were included in the analysis. The filtered list of PAMs was used to generate a sequence logo using Logomaker^48^.

### In vitro DNA cleavage with purified protein

Purified Cas9d-MG34-1 was prepared with Luria-Bertani (LB) medium starter culture inoculated with fresh plate scraped co-transformants in BL21 (DE3) containing expression plasmids, which carry T7-driven *cas9d-MG34-1-6XHis* and sgRNA. The culture was incubated at 37 °C overnight, then transferred to a larger autoinducing medium culture incubated at 30 °C for 7 h, cooled to 18 °C, and shaken for 16 h. After cell harvest, cells were resuspended in a lysis buffer containing storage buffer (20 mM Tris-HCl pH 7.5, 300 mM NaCl, 5% glycerol, and 10 mM MgCl_2_), EDTA-free protease inhibitor (Thermo Fisher Scientific), and 10 mM imidazole and then lysed via sonication. The lysate was loaded on a HisTrap FF (Cytiva) column and the protein was eluted with an isocratic step of 250 mM imidazole in the storage buffer. Peak fractions were pooled after analysis on SDS PAGE and the buffer was exchanged into the storage buffer using Zeba desalting columns (Thermo).

For guide length testing, RNP complexes of Cas9d-MG34-1 were assembled by pre-incubating the purified enzyme with a previously in vitro transcribed sgRNA with spacer lengths ranging from 16 to 30 nucleotides. The complex was incubated at a 1.5:1 sgRNA:effector ratio for 5 min at 25 °C in 1x Effector Buffer (10 mM Tris-HCl pH 7.5, 100 mM NaCl, and 0 mM MgCl_2_). Cleavage reactions were performed by adding the previously prepared RNP samples to 50 nM DNA in a 35:1 RNP:DNA ratio in 1x Effector Buffer. The reactions were incubated at 37 °C for 2 h and then quenched by adding 0.2 ug of RNAse A (New England Biolabs) and incubation at 37 °C for 10 min, then subsequent addition of 4 units of proteinase K (New England Biolabs) and incubation at 55 °C for 10 min. DNA Loading dye (6x, New England Biolabs) was added, and all reactions were analyzed by gel electrophoresis using a 1.5% agarose gel stained with gel green (Biotium). DNA bands were visualized by a Chemi-Doc imager (Biorad) and band intensities were quantified using BioRad Image Lab v6.0. Successful cleavage results in the 500 bp DNA being split into two fragments of 150 and 350 bp.

### In vitro spacer length optimization for HEARO MG35-1 and Cas9d-MG102-2

Nucleases were expressed using in vitro transcription/translation (New England Biolabs) at 37 °C for 2 h. Transcription was driven by a T7 promoter on a linear DNA template coding for the nuclease, which was combined with 3 uM of in vitro transcribed sgRNA designed with spacer lengths ranging from 14 to 28 nt. In vitro cleavage reactions were performed by adding the RNP samples to 5 nM supercoiled DNA in a 1:5 v/v ratio in 1x Effector Buffer (10 mM Tris-HCl pH 7.5, 100 mM NaCl, and 10 mM MgCl_2_) for Cas9d-MG102-2, and 1x New England Biolabs 2.1 buffer (10 mM Tris-HCl pH 7.9, 50 mM NaCl, 10 mM MgCl_2_, and 100 µg/ml BSA) for MG35-1. The reactions were incubated at 37 °C for 2 h and then quenched by adding 0.2 ug of RNAse A (New England Biolabs) and incubation at 37 °C for 20 min, then subsequent addition of 4 units of proteinase K (New England Biolabs) and incubation at 55 °C for 30 min. Reactions were analyzed by capillary electrophoresis using a D5000 Tapestation kit (Agilent) following the instructions recommended by the manufacturer for analysis and visualization. Successful cleavage results in the supercoiled 2200 bp DNA being cut into linear dsDNA.

### Plasmid interference in *E. coli*

For testing of nuclease activity in bacterial cells, *E. coli* BL21 (DE3) strains (New England Biolabs) were transformed with plasmids containing T7 or ptac-driven effector (ampicillin resistance) and T7-driven sgRNA (chloramphenicol resistance) (10 ng each plasmid), plated, and grown overnight. The resulting colonies were cultured overnight in triplicate, then subcultured in LB with antibiotics and grown to OD 0.4–0.6. A 1.25 OD equivalent of cell culture was made competent according to standard protocols (Zymo Mix and Go kit) and transformed with 100 ng of a kanamycin plasmid, either with or without a target spacer and PAM in the backbone. After heat shock, transformations were recovered in SOC for 2 hr at 37˚C. Nuclease efficiency was determined by a five-fold dilution series grown at 37 ˚C overnight on induction media (LB agar plates with antibiotics and 0.05 mM IPTG). In the presence of the antibiotic, the effectors that successfully target and cut the antibiotic resistance plasmid result in growth repression. Colonies were quantified from the dilution series to measure overall repression due to nuclease-driven plasmid cleavage.

### Mismatch plasmid interference in *E. coli*

For testing mismatch specificity in bacterial cells, *E. coli* BL21 (DE3) strains (New England Biolabs) were transformed with plasmids containing T7-driven effector (ampicillin resistance) and T7-driven sgRNA (chloramphenicol resistance), plated, and grown overnight. The resulting colonies were made competent as described above and transformed with 100 ng of a kanamycin plasmid in three conditions: a target spacer and PAM in the backbone, a library of 25 plasmids, each containing a single mismatch along a 24 nt spacer and constant PAM, or a control plasmid with no spacer or PAM. After heat shock, transformations were recovered in SOC for 2 h at 37 ˚C. Cultures were plated and grown at 37 ˚C overnight on induction media (LB agar plates with antibiotics and 0.05 mM IPTG). Plasmids were extracted from the surviving colonies via miniprep (Qiagen). The target region was amplified via PCR and analyzed via NGS. Enriched spacers relative to the untreated library were unable to be recognized and cut by the nucleases, and, thus, are considered to be regions where the effectors do not tolerate a mismatch.

### Base editing in *E. coli*

Base editing efficiency in *E. coli* was assessed as follows: 1 μL plasmid with a concentration of 10 ng/μL was transformed into 25 μL BL21 (DE3) electrocompetent cells (Lucigen) and recovered in 475 μL recovery expression media in a 96-well deep well plate at 37 °C for 1 h. About 100 μL of resulting cells were plated on LB agar plates containing 100 μg/mL ampicillin and 0.1 mM IPTG and incubated at 37 °C for 18 h. Twelve colonies were picked, and the lacZ gene was amplified by Q5 DNA polymerase (New England Biolabs). The resulting PCR products were purified and sequenced by Sanger sequencing (Elim Biopharmaceuticals, Inc). Base edits were determined by examining whether cytosines were converted to thymines for cytosine base editing, or adenines were converted to guanines for adenine base editors in the targeted regions. Editing efficiency was calculated as follows: (number of edited colonies/number of total colonies) × 100. The results show averaged editing efficiencies from two independent experiments.

### Base editing *E. coli* positive selection

MG35-1 adenine base editor (ABE) activity was tested with nickase MG35-1 (D59A mutation) with either an N-terminal or a C-terminally fused TadA*(7.10) monomer and a C-terminus SV40 NLS. This ABE was tested with sgRNA containing a 20 bp spacer sequence either targeting the chloramphenicol acetyltransferase (CAT) gene or a non-targeting spacer sequence of the same 20 nucleotides in a scrambled order. The CAT gene contains an H193Y mutation that renders it nonfunctional for chloramphenicol resistance. The ABE, sgRNA, and nonfunctional CAT gene were cloned into a pET-21 backbone containing Ampicillin resistance. We transformed 10 ng of the plasmid into 25 μL of BL21(DE3) (Lucigen) *E. coli* cells and incubated them with shaking at 37 °C in 450 uL of recovery media for 90 min. We plated 70 μL of media onto plates containing chloramphenicol concentrations of 0, 2, 3, 4, and 8 μg/mL. The 0 μg/mL plate was used as a transformation control. Plates also contain 100 μg/mL carbenicillin and 0.1 mM IPTG. Plates were incubated at 37 °C for 40 h. CAT mutations were verified in the resulting colonies by Sanger sequencing (Elim Biopharmaceuticals, Inc).

### Base editing in human cells

HEK293T cells were purchased from ATCC. The cells were authenticated by the provider using STR profiling. Cells were grown and passaged in Dulbecco’s Modified Eagle’s Medium plus GlutaMAX (Gibco) supplemented with 10% (v/v) fetal bovine serum (Gibco) at 37 °C with 5% CO_2_. In total, 2.5 × 10^4^ cells were seeded on 96-well cell culture plates treated for cell attachment (Costar) and grown for 20 to 24 h (spent media were refreshed with new media before transfection). Each plate well received 300 ng expression plasmid and 1 μL lipofectamine 2000 (Thermo Fisher Scientific) for transfection, as per the manufacturer’s instructions. Transfected cells were grown for 3 days, harvested, and genomic DNA was extracted with QuickExtract (Lucigen) per the manufacturer’s instructions. Targeted regions for base edits were amplified using Q5 High-Fidelity DNA polymerase (New England Biolabs) with target-specific primers and PCR products were purified with the HighPrep PCR Clean-up System (MAGBIO) per the manufacturer’s instructions. To analyze base editing, adapters used for next-generation sequencing (NGS) were appended to PCR products by subsequent PCR reactions using the KAPA HiFi HotStart ReadyMix PCR Kit (Roche) and primers compatible with TruSeq DNA Library Prep Kits (Illumina). DNA concentrations of the resulting products were quantified by TapeStation (Agilent), and samples were pooled together to prepare the library for NGS analysis. The resulting library was quantified by qPCR with the Aria Real-time PCR System (Agilent), and high throughput sequencing was performed with an Illumina Miseq instrument per the manufacturer’s instructions. Sequencing data were analyzed for base edits by CRISPResso2^49^.

### mRNA synthesis

The CDS codifying for Cas9d-MG34-1 with a 3xFLAG epitope in the C-terminus was cloned into a pUC19 plasmid. An RNA-pol T7 promoter along with 5′ and 3′ UTRs and a 100 nt polyA tail were included. About 100 µg of plasmid was digested with SapI to linearize it downstream of the polyA tail. The plasmid was subsequently purified with phenol/chloroform and precipitated with 70% ethanol. The DNA pellet was resuspended in 20 µl of nuclease-free water. For in vitro transcription, 1 µg of linearized plasmid DNA was added to a 20 µl reaction containing 1X reaction buffer (40 mM Tris-HCl pH 7.5, 16.5 mM MgCl_2_, 50 mM NaCl, 2.5 mM Spermidine, and 1 mM DTT) and 750 units of Hi-T7 RNA Polymerase (New England Biolabs). The reaction was incubated at 50 °C for 1 h. Recently transcribed mRNA was purified using MEGAclear transcription Clean-up kit (Thermo Fisher Scientific) following the manufacturer’s instructions.

### Immunofluorescence in HEK293T cells

The day prior to transfection, HEK293T cells were seeded at 70,000 cells per well in 24 well plates on 12 mm round coverslips (Corning). On the day of transfection, 300 ng of mRNA codifying for Cas9d-MG34-1 (3XFLAG) was complexed in Lipofectamine Messenger Max (Thermo Fischer Scientific) following the manufacturer’s instructions and the complex was added to cells. Twenty-four hours post-transfection, cells were fixed by adding 4% formaldehyde (Sigma Aldrich) for 20 min at room temperature. Subsequently, cells were washed three times with PBS and permeabilized/blocked by adding blocking buffer (0.1% TX-100 in 2% FBS) for 20 min. Cells were then incubated with 1:100 dilution of anti-FLAG (Sigma Aldrich, F3165), in blocking buffer for 1.5 h at room temperature, extensively washed and incubated in secondary antibodies diluted 1:1000 in blocking buffer and Alexa Fluor 488 (Thermo Fischer Scientific, A32723) for 1.5 h. Cells were extensively washed, rinsed briefly in dH_2_O and mounted on slides with ProLong Gold with DAPI (Thermo Fischer Scientific). For antibody validation experiments, non-transfected HEK293T cells were imaged, keeping all the settings constant in an EVOS 5000 (Thermo Fisher Scientific).

### Gene editing in the TRAC locus

K562 cells were purchased from ATCC and cultured according to ATCC protocols. Two sgRNA targeting the TRAC locus were designed based on the MG102-2 PAM and chemically synthesized by IDT. For gene editing experiments, 500 ng of in vitro synthesized MG102-2 mRNA and either 150, 300, or 450 pmol of the indicated sgRNA were co-nucleofected in 1.5E5 cells using the Lonza 4D Nucleofector (program FF-120). In parallel, cells were nucleofected with neither mRNA nor guide to assessing background at sites targeted by TRAC guides (mock). Cells were harvested 72 h post-electroporation for genomic DNA extraction using QuickExtract (Lucigen #09050) and processed for amplicon next-generation sequencing on an Illumina Miseq as described above. The resulting data were analyzed with an in-house indel calculator script.

### Reporting summary

Further information on research design is available in the Nature Portfolio Reporting Summary linked to this article.

## Supplementary information

Supplementary Information

Reporting Summary

Description of Additional Supplementary Files

Supplementary Data File 1

Supplementary Data File 2

Supplementary Data File 3

Supplementary Data File 4

## Data Availability

The data generated in this study, including Cas9d, Cas9c2, Cas1, and HEARO protein sequences and active sgRNA sequences are available as Supplementary Materials. NCBI accession numbers for sequences generated here include: OP541498, OP541499, OP541500, OP541501, OP541502, OP541503, OP541504, OP541505, OP541506, OP541507, OP541508, OP541509, OP541510, OP541511, OP541512, OP541513, OP541514, OP541515, OP541516, OP541517, OP541518, OP541519, OP541520, OP541521, OP541522, OP541523, OP541524, OP541525, OP541526, OP541527, OP541528, OP541529, OP541530, OP541531, OP541532, OP541533, OP541534, OP541535, OP541536, OP541537, OP541538, OP541539, OP541540, OP541541, OP541542, OP541543, OP541544, OP541545, OP541546, OP541547, OP541548, OP541549, OP541550, OP541551, OP541552, OP541553, OP541554, OP541555, OP541556, OP541557, OP541558, OP541559, OP541560, OP541561, OP541562, OP541563, and OP541564.
