# Supplementary material for: Compact Cas9d and HEARO enzymes for genome editing discovered from uncultivated microbes
Source: Nat Commun. 2022 Dec 15;13:7602. doi: 10.1038/s41467-022-35257-7 (PMC9755519; doi:10.1038/s41467-022-35257-7)
Supplement: Supplementary file 1 — Supplementary Information [file 41467_2022_35257_MOESM1_ESM.pdf]

Supplementary Information

Supplementary Tables

Supplementary Data File 1. Protein sequences and taxonomic classification for SMART effectors recovered in this work. Metagenomic sequencing accession numbers are included (Excel).

Supplementary Data File 2. Amino acid composition of SMART nucleases (Excel).

Supplementary Data File 3. Active SMART system information (Excel).

Supplementary Data File 4. Genome binning information for active SMART systems (Excel).

Supplementary Table 1. Colony counts for *E. coli* survival assay with ABE-MG35-1. Colonies grown on plates containing chloramphenicol concentrations of 0, 2, 3, and 4 ug/mL were sequenced to confirm reversion of the CAT gene function. Experiments were performed as n=2.

	**Edited colonies**	
**Chloramphenicol (ug/mL)**	**Target spacer**	**Non-target spacer**
0	1 / 10	0 / 10
2-4	26 / 30	No colonies
8	No colonies	No colonies

Supplementary Figures

Supplementary Figures S1 through S19

Supplementary Figures

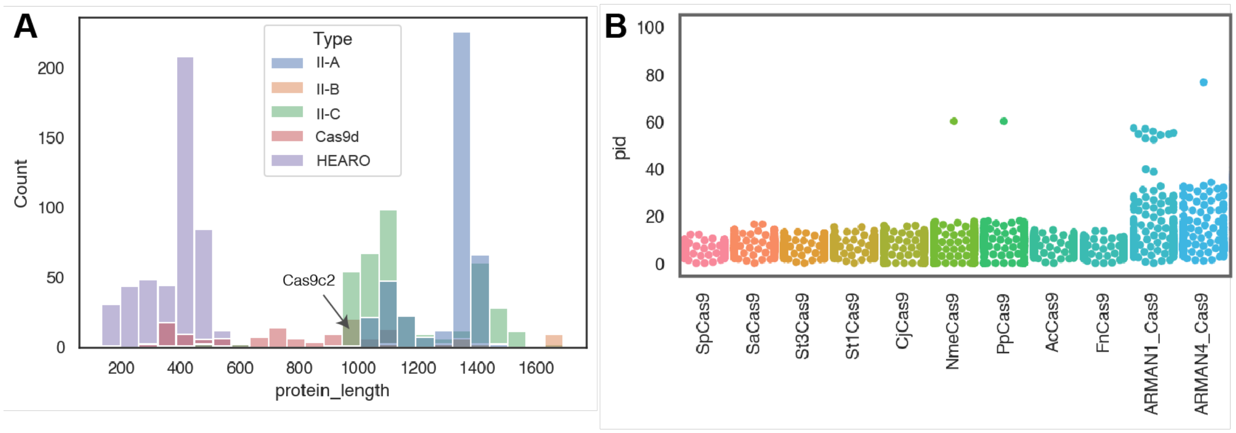

Supplementary Figure 1. Summary statistics of nuclease effectors reported here vs. Cas9 references.

A) Count distribution of Cas9c2, Cas9d and HEARO nucleases of varying length show a bimodal distribution with one peak around 400 aa (HEARO, purple bars) and a second peak around 750 aa (Cas9c2 and Cas9d, pink bars). Cas9 nucleases of the II-A (blue bars), II-B (orange bars), and II (green bars) C also show a bimodal distribution with peaks around 1,100 aa (e.g. SaCas9) and 1,300 aa (e.g. SpCas9).

B) Distribution of percent identity of predicted Cas9c2 and Cas9d enzymes vs. selected reference Cas9 sequences. ARMAN4_Cas9 and ARMAN1_Cas9 derived from Archaea [1] and are the only two members of the type II-C2 subclass.

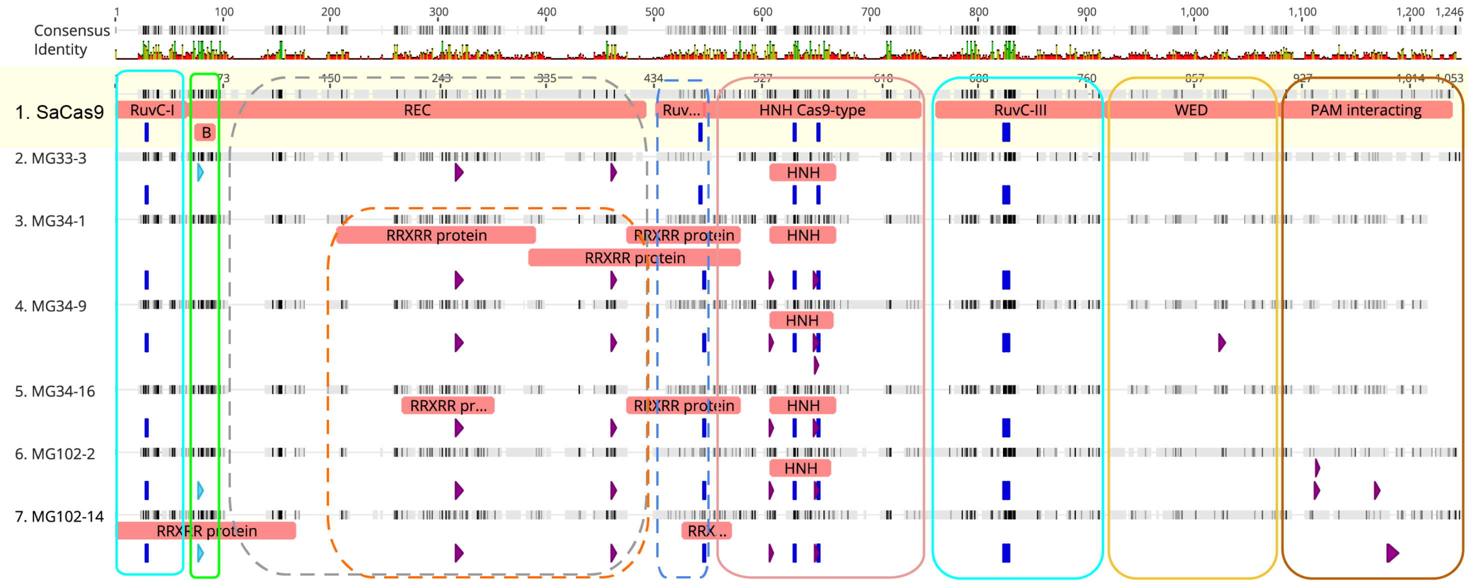

Supplementary Figure 2. Domain architecture of Cas9c2 and Cas9d nucleases represented on a multiple sequence alignment vs. the reference SaCas9 sequence. Annotations below each sequence are as follows: RuvC and HNH catalytic residues (blue bars); Zn-binding ribbon motifs (purple arrows); and RRXRR amino acid motifs (light blue arrows). Regions that align in 3D space with the crystal structure of SaCas9 are represented by closed boxes on the alignment. Dashed lines represent regions with poor or no alignment in 3D space between the 3D structure prediction of Cas9d and SaCas9.

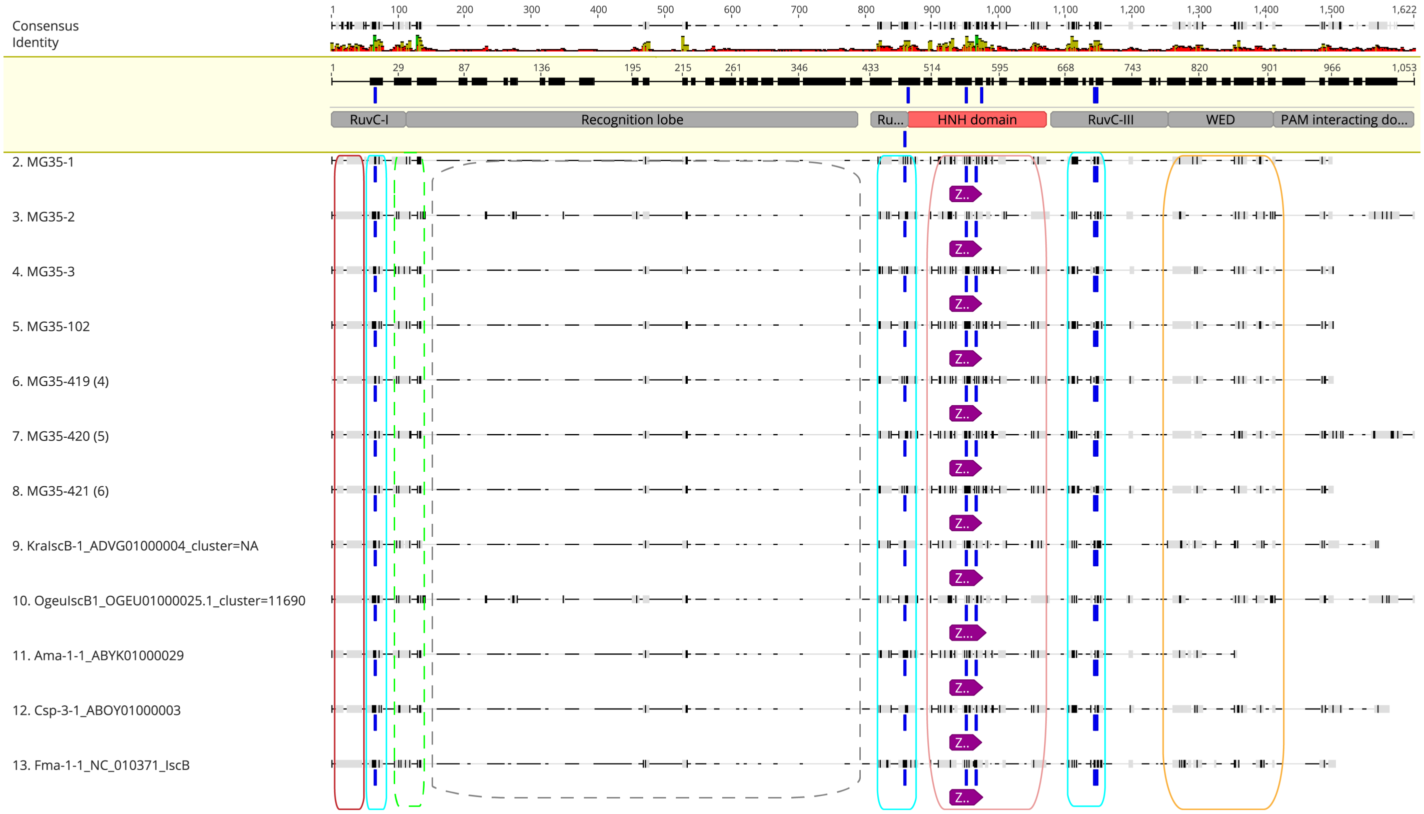

Supplementary Figure 3. Multiple sequence alignment of seven active HEARO nucleases vs. a reference SaCas9 nuclease sequence. Reference IscB (KraIscB-1 and OgeuIscB1) [2] and HEARO ORF (Ama-1-1, Csp-3-1, and Fma-1-1) [3] sequences were included. Annotations below each sequence: RuvC and HNH catalytic residues (blue bars); Zn-finger (purple arrows). Domains shared with the SaCas9 [4] and OgeuIscB1 [5] structures are represented by closed boxes on the alignment. Dashed lines represent regions with poor or no alignment in 3D space with the SaCas9 structure.

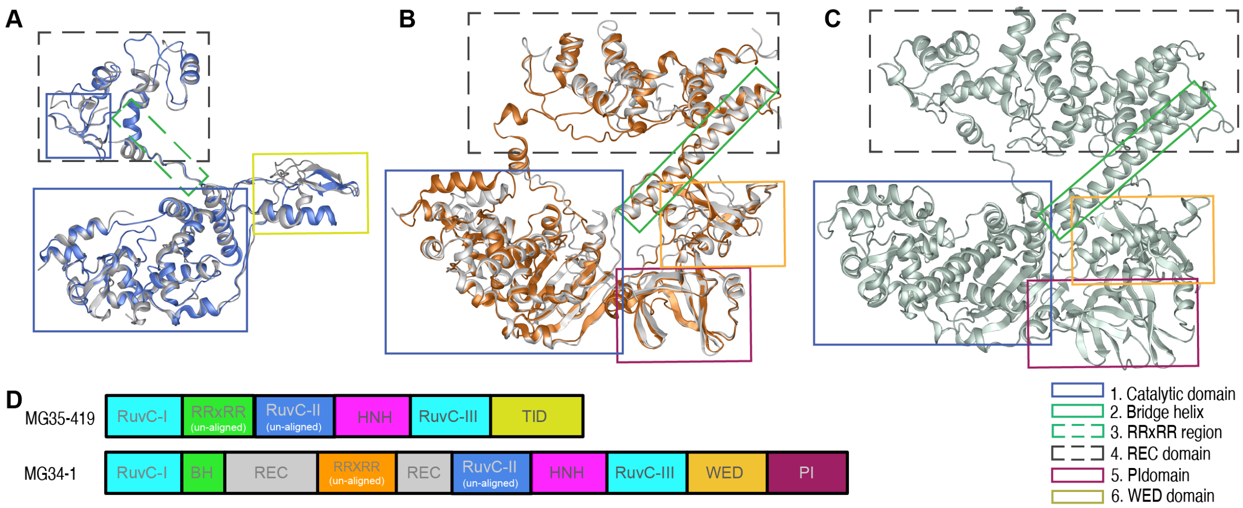

Supplementary Figure 4. 3D structure prediction for Cas9d and HEARO enzymes. Domains shared with the reference *Staphylococcus aureus* SaCas9 structure are highlighted in colored squares.

A) 3D modeling of the HEARO nuclease MG35-419 (blue chain) vs. SaCas9 (grey chain).

B) 3D modeling of the Cas9d-MG34-1 nuclease (orange chain) vs. SaCas9 (grey chain).

C) 3D structure of the reference SaCas9 enzyme, downloaded from the Protein Data Bank database (accession number 5AXW).

D) Predicted domain architecture based on sequence similarity and 3D modeling for MG35-419 and Cas9d-MG34-1. BH: bridge helix; REC: recognition lobe; WED: wedge domain; PI; PAM interacting domain; TID: TAM interacting domain.

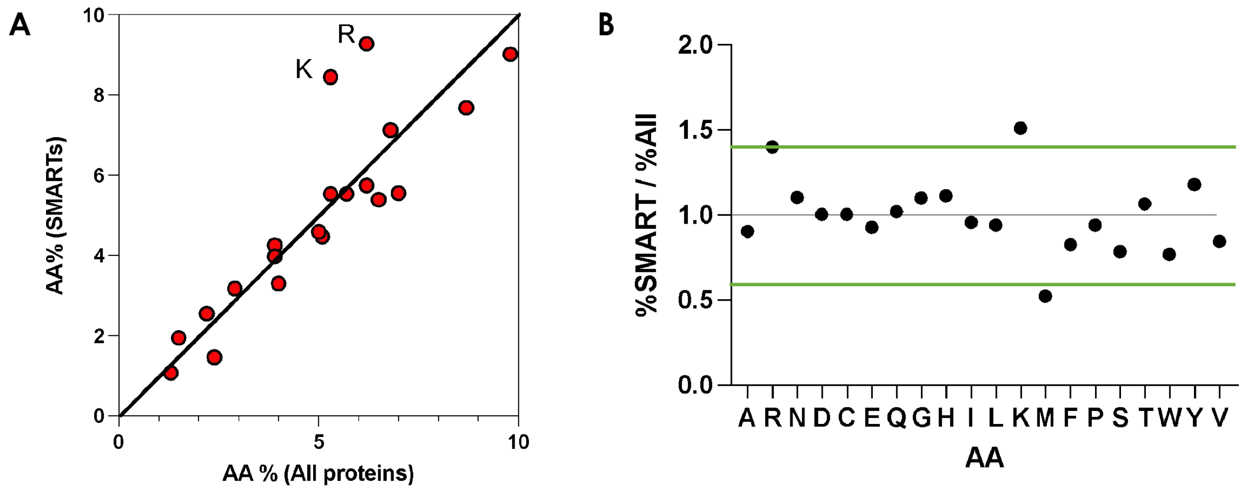

Supplementary Figure 5. Average amino acid content of Cas9d deviates from typical proteins.

A. Scatterplot of the average amino acid content of proteins in the Uniref50 database analyzed by Carugo, 2008 [6] (X axis) vs. the percentage of amino acid content in Cas9d proteins (Y axis). The arginine (R) and lysine (K) content deviates from the linear trend.

B. Graph showing the ratio of amino acid percentages in Cas9d proteins to the percentages in the Uniref50 database. The mean of all ratios is 0.99, with SD 0.22. Green lines show two standard deviations from the average, assuming normality.

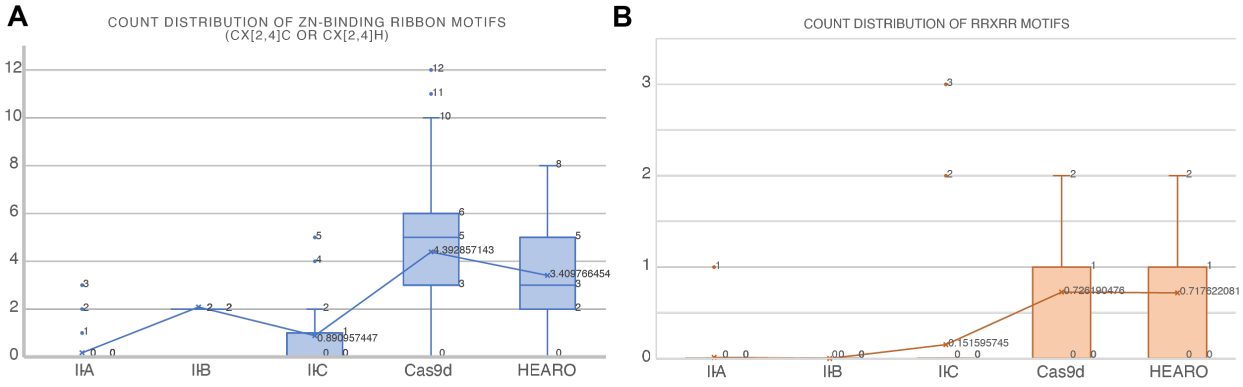

Supplementary Figure 6. Number of specific motifs present in Cas9d and HEARO nucleases relative to motifs predicted in Cas9 reference sequences. Zn-binding ribbon (A) and RRxRR (B) motifs were predicted on 803 reference Cas9 sequences (type II-A, II-B, and II-C) and 555 nuclease sequences reported here. Lines track the mean count value, outliers are represented by dots, and mean, median and maxima values are shown.

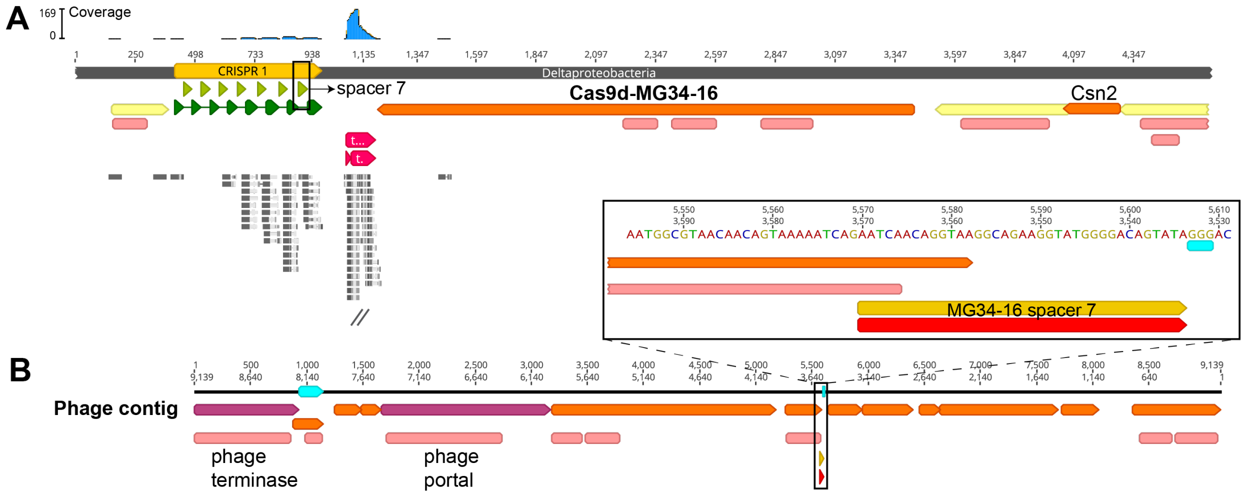

Supplementary Figure 7. Cas9d CRISPR systems are active anti-viral systems in nature.

A) Genomic context of the nuclease Cas9d-MG34-16. Environmental expression sequencing reads are shown aligned under the CRISPR array and the predicted tracrRNA, and the transcriptomic coverage for the regions is illustrated above the contig sequence. Cas9d-MG34-16 associated CRISPR spacer 7 (delineated by a black box) was identified as having a perfect match targeting a phage genomic fragment.

B) Genomic fragment targeted by spacer 7 from the Cas9d-MG34-16 CRISPR array. The genomic fragment was identified as being derived from phage based on virus-specific “terminase” and “portal” protein annotations. Inset shows the location of the Cas9d-MG34-16 spacer 7 targeting the C-terminus of a viral gene of unknown function. The putative 3’ NGG PAM is highlighted by a teal box downstream from the spacer match.

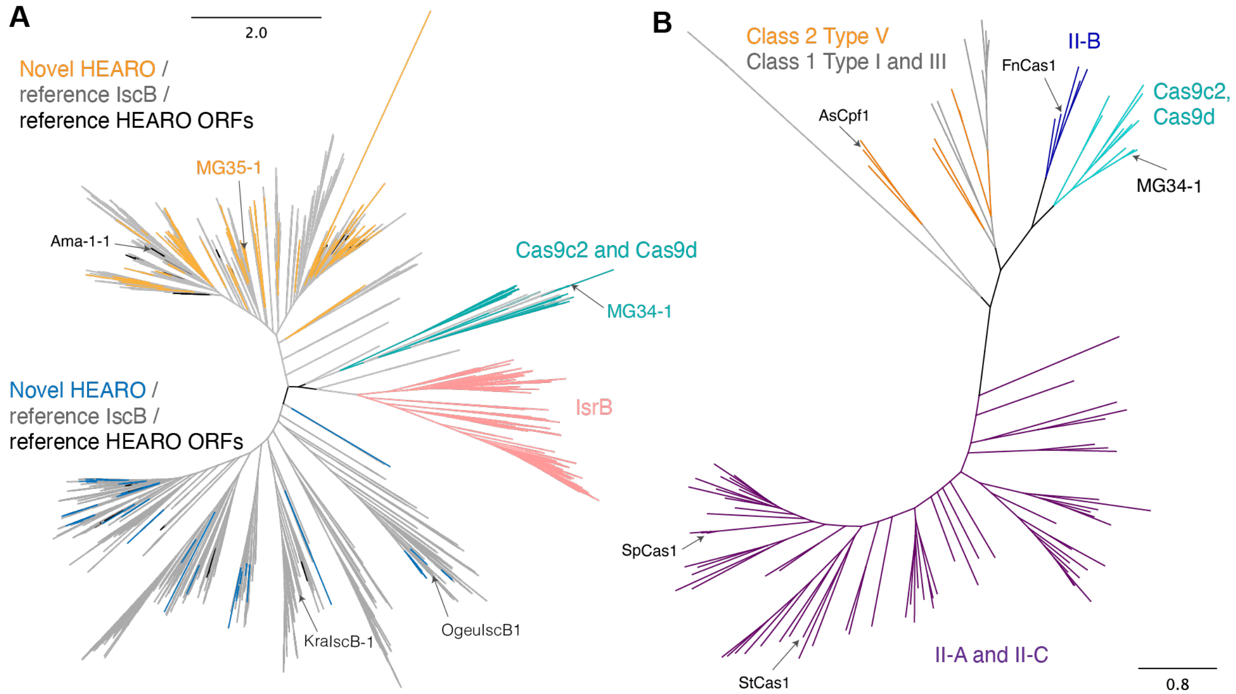

Supplementary Figure 8. Phylogenetic tree for nucleases reported (A) and, when applicable, their associated Cas1 genes (B). The trees were inferred from a multiple sequence alignment of full-length Cas1 protein sequences with FastTree2.

A) Cas9c2, Cas9d and HEARO nucleases phylogenetic tree. IscB (grey branches) and HEARO ORF (black branches) reference sequences were included. HEARO and IscB sequences separate into two distinct clades (bottom and top clades). Reference HEARO ORF Ama-1-1 [3] and IscB (KraIscB-1 and OgeuIscB1) [2,5] are labeled.

B) Cas9c2 and Cas9d systems are distantly related to CRISPR Class 2 type II-B and Class 1 systems. Reference Cas1 sequences associated with Class 2 Type II-A, II-B, II-C and Type V systems, as well as with Class 1 Type I and III systems were included in the phylogenetic tree.

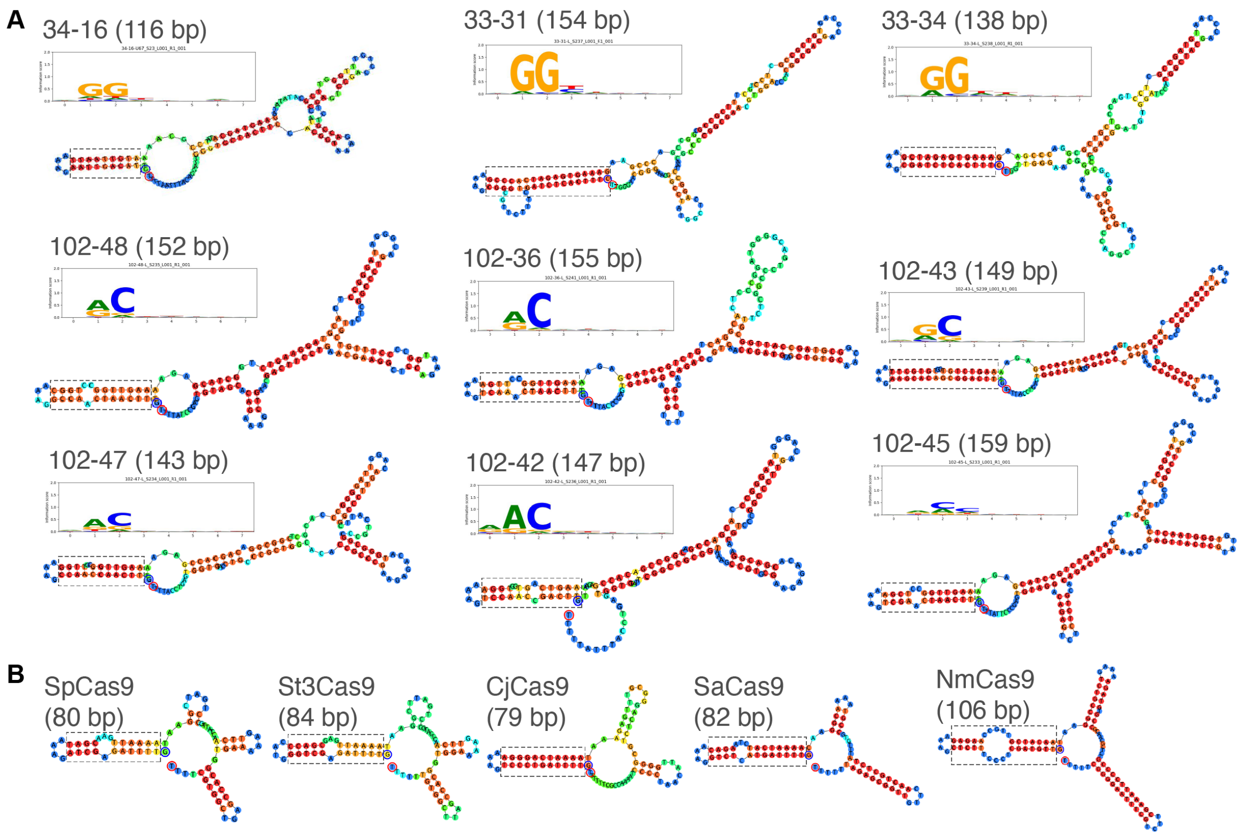

Supplementary Figure 9. Engineered single guide RNA designs for active Cas9c2 and Cas9d (A) and reference Cas9 nucleases (B). Hairpin highlighted by a dashed box represents the repeat-antirepeat sequences joined by a tetraloop. The targeting spacer is added to the 5’ end of the sgRNAs (in the structures, the 5’ most base is highlighted by a blue/black circle). Reference sgRNAs were folded from sequences reported for SpCas9, St3Cas9, SaCas9 and NmeCas9 by Nowak et al [7], and for CjCas9 by Kim et al [8].

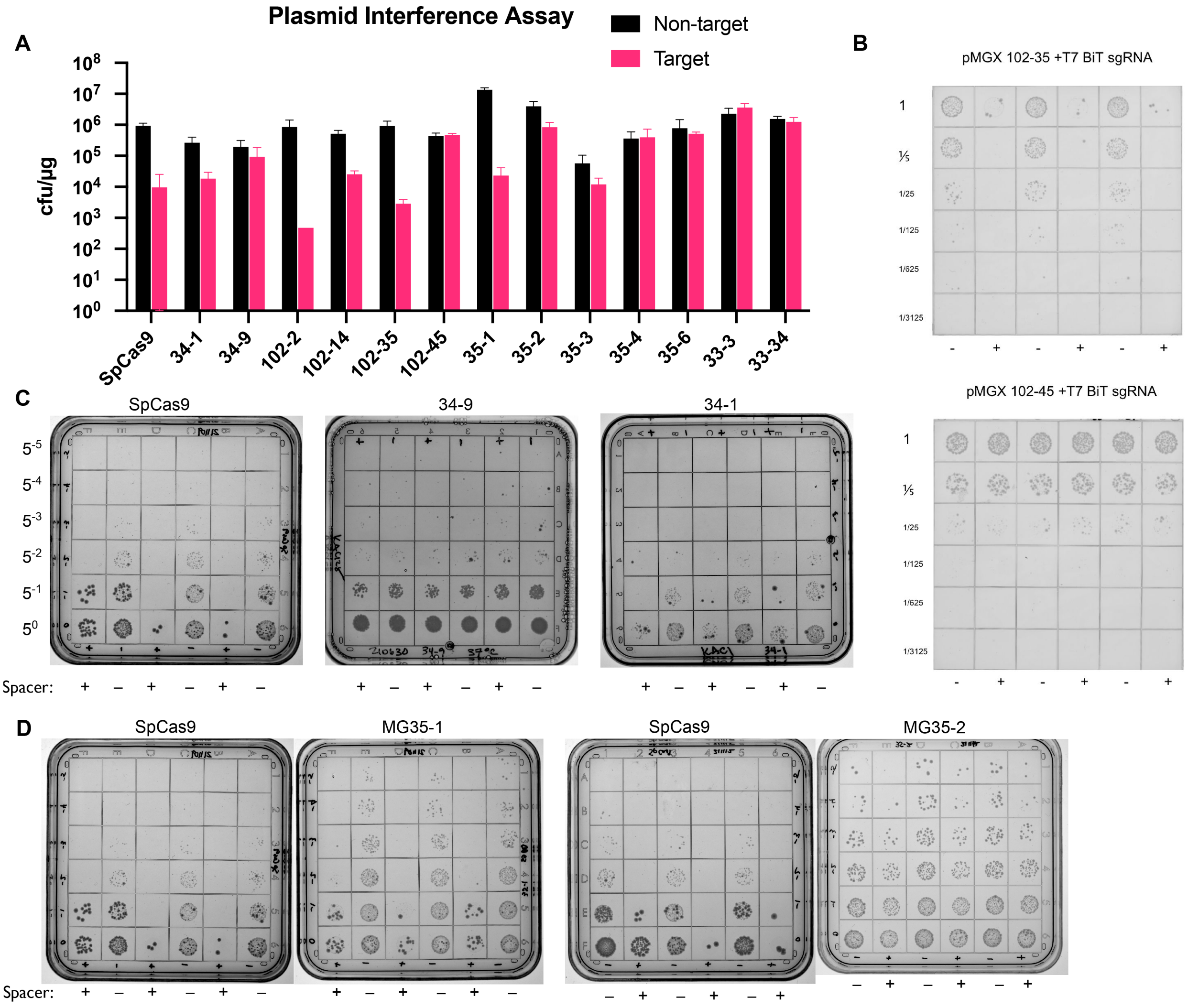

Supplementary Figure 10. Plasmid targeting experiments demonstrate nuclease activity in *E. coli.*

A) Bar plot of colony forming unit (cfu) measurements (in log-scale) showing *E. coli* growth repression in the target condition vs. the non-target controls. Plasmid interference assays for each nuclease were done in triplicate along with the SpCas9 positive control. Error bars represent the standard deviation from the mean.

B-D) Strains expressing the positive control SpCas9, Cas9d (B-C) or HEARO (D) enzymes, as well as their corresponding sgRNA were transformed with a kanamycin resistance plasmid containing a target for the sgRNA (+ columns on each plate). Plate quadrants that show

growth impairment (+) vs. the negative control without the target and PAM (-) indicate successful targeting and cleavage by the enzyme. The experiments were performed in triplicate.

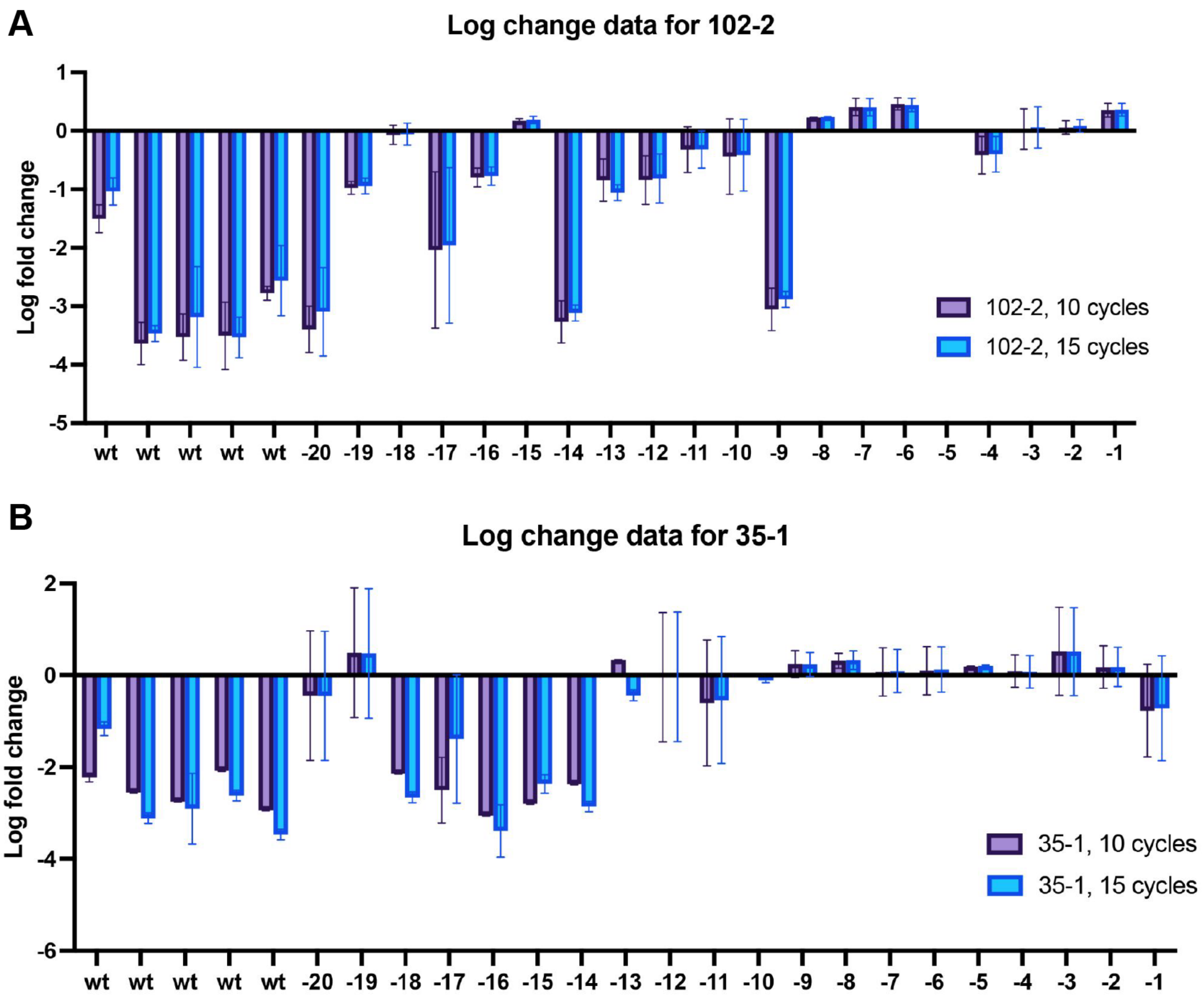

Supplementary Figure 11. Mismatch plasmid interference assays show the log fold change cleavage activity for spacers with mismatches at each position of the tested spacer for Cas9d-MG102-2 (A) and HEARO MG35-1 (B). Error bars represent the standard deviation from the mean.

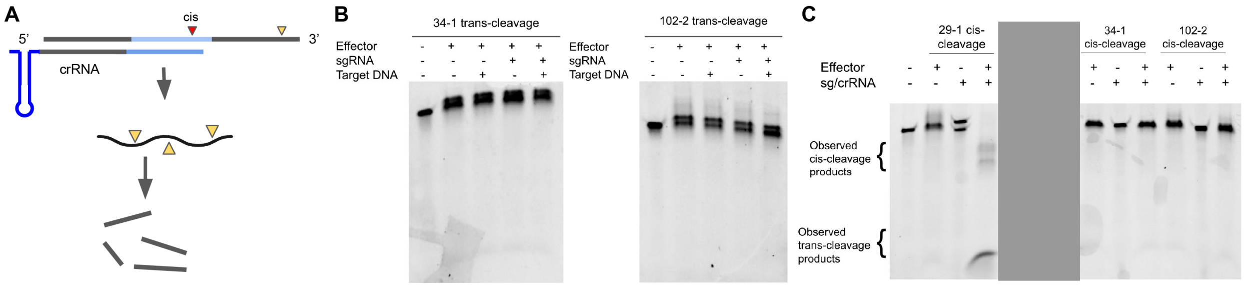

Supplementary Figure 12. Cas9d nucleases do not exhibit cleavage activity on ssDNA.

A) Reactions for ssDNA cleavage were assayed on 6-FAM labeled ssDNA (top line) incubated with effector and guide RNA, performed at 37°C for 1 hour and imaged with a gel green channel. Cis- (red triangle) or trans- (yellow arrows) cleavage activity will produce fragments that migrate lower on the gel.

B) Gel showing trans-cleavage activity results for Cas9d-MG34-1 and Cas9d-MG102-2. Band at the top of the gel represents the target ssDNA. Absence of bands on lane 5 (reaction with both effector and guide RNA) indicate lack of trans-cleavage activity.

C) Gel showing cis-cleavage activity results for Cas9d-MG34-1 and Cas9d-MG102-2. Band at the top of the gel represents the target ssDNA. A Type V-A nuclease (MG29-1 [9]) that exhibits cis-cleavage activity was included as positive control. Absence of bands on lanes 11 and 14 (reaction with Cas9d effectors and guide RNA) indicate lack of trans-cleavage activity. Lanes 5-8 are not relevant for this assay and were greyed out. Experiments were done once.

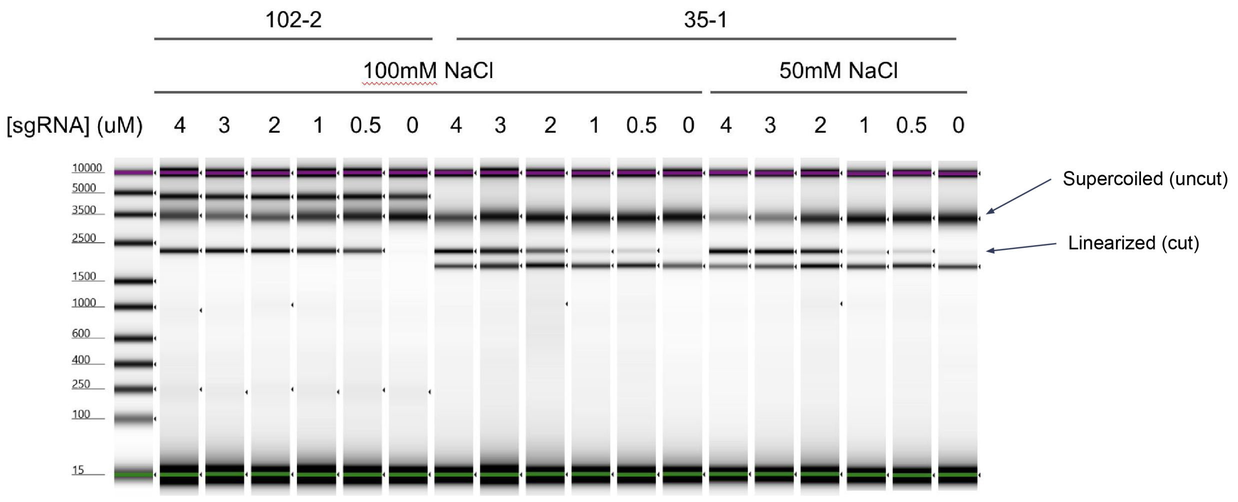

Supplementary Figure 13. Single guide and salt concentration titration for active nucleases.

In vitro cleavage assays for Cas9d-MG102-2 (lanes 1-6) and HEARO MG35-1 (lanes 7-18) show cleavage of target plasmid DNA (at ~3500 bp) into linear DNA products (smaller than 2500 bp). Darker “linearized” bands indicate higher cleavage efficiency.

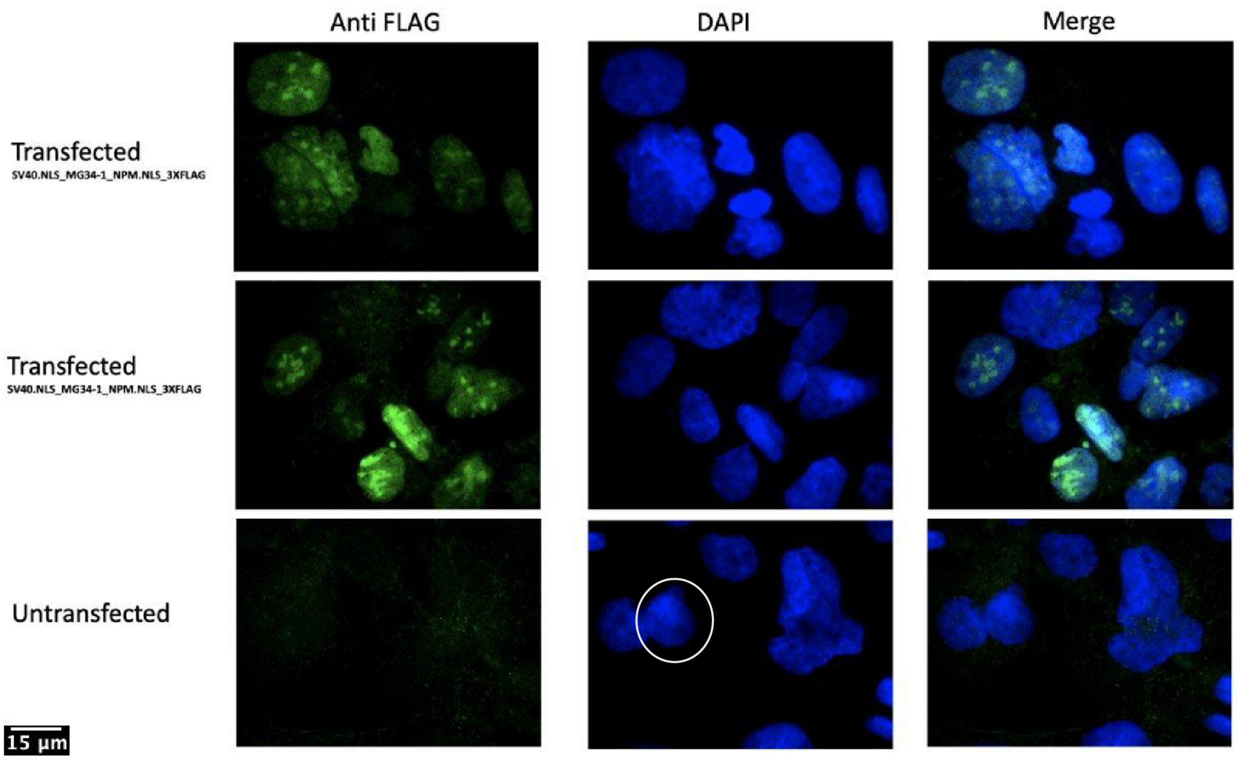

Supplementary Figure 14. Immunofluorescence staining shows Cas9d-MG34-1 localizes in the nucleus of mammalian cells. Cas9d-MG34-1 was tagged with a 3X-FLAG in the C terminus. Anti-FLAG antibody was used for Cas9d-MG34-1 detection. The specificity of the antibody is shown by absence of signal in the untransfected control. Experiments were repeated twice. The scale bar represents the diameter of the white circle around one nucleus.

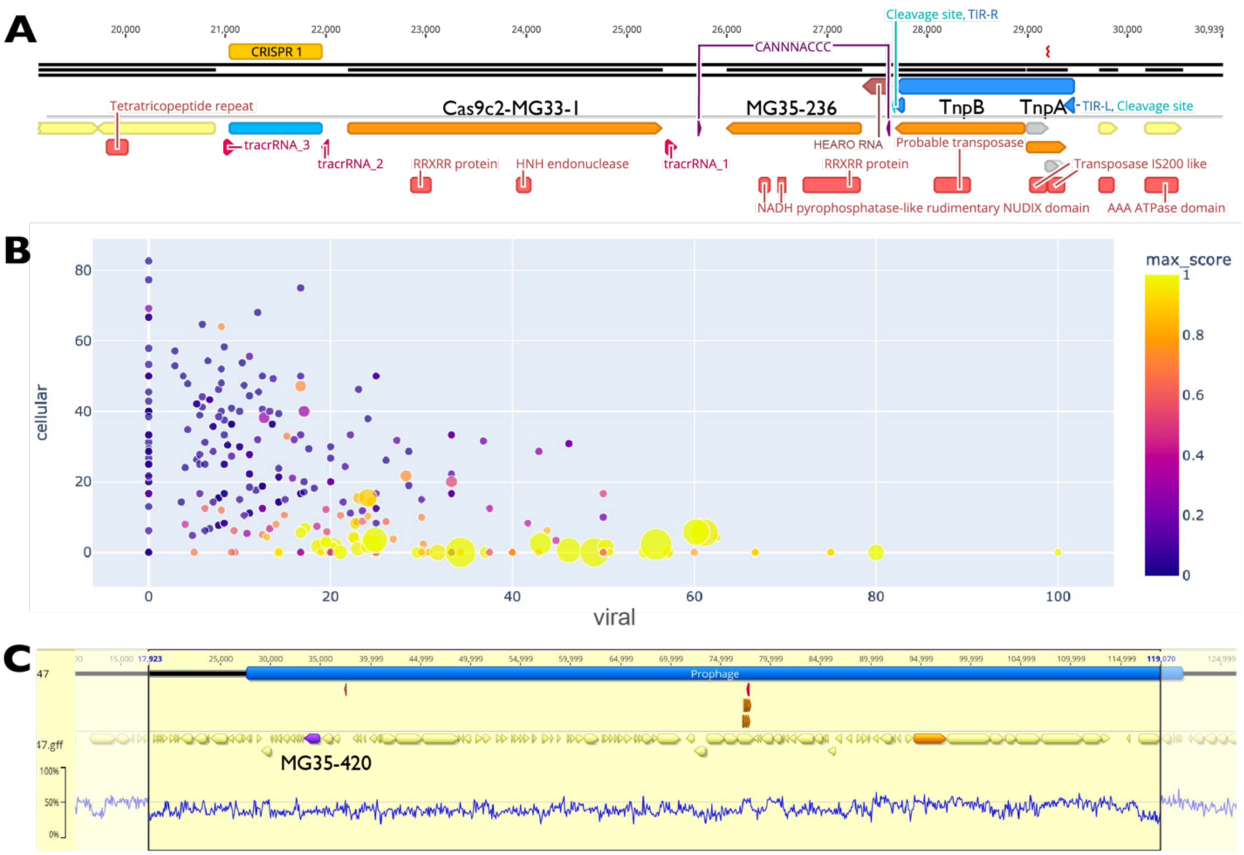

Supplementary Figure 15. HEARO effectors are associated with viral genomes.

A) Genomic fragment recovered which encodes a HEARO enzyme (MG35-236) downstream from a transposon IS200/IS605 (blue arrow). Putative TnpA binding sites (CANNNACCC) flank the HEARO gene and predicted RNA. Intergenic regions containing CRISPR anti-repeats were annotated as potentially encoding tracrRNAs for the Cas9c2-MG33-1 system.

B) Distribution of HEARO effector genomic fragment classifications. Over 300 contigs are represented in the plot. Plots illustrate the ratio of genes in each contig classified as viral (x-axis) and non-viral ("cellular", y-axis) based on HMM profile searches. Each dot represents a HEARO encoding contig and is colored by score (scores >.9 are typically classified as viral in metagenomics analyses). The size of each dot represents the number of "hallmark" viral genes detected in that contig.

C) Example prophage encoding the active HEARO nuclease MG35-420. The prophage boundaries were predicted with Virsorter v2 and overlap a region with lower GC skew than the rest of the contig.

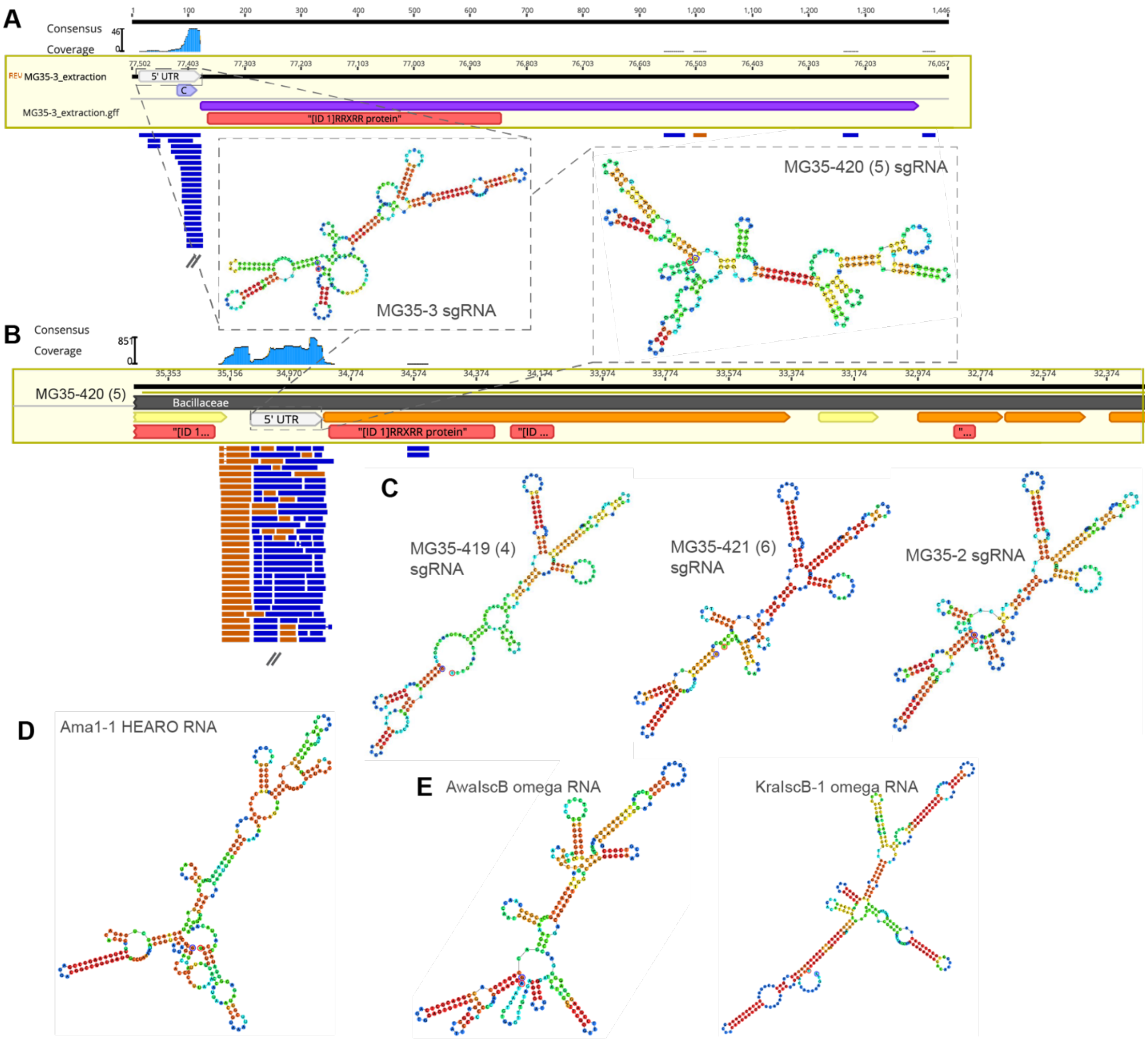

Supplementary Figure 16. HEARO RNAs are encoded in the system's 5' UTR.

A) Genomic context of the HEARO MG35-3 effector showing environmental RNA transcriptomics reads mapping upstream from the start codon (5' UTR) in the forward orientation. Predicted Pfam domains are represented by rectangles below arrows, and its predicted guide RNA is annotated upstream from the nuclease. The HEARO RNA design is shown as inset.

B) Genomic context of the HEARO MG35-420 effector showing RNA transcriptomic reads sequenced from an in vitro transcription reaction of the effector with its native 5' UTR.

Predicted Pfam domains are represented by rectangles below arrows, and its predicted guide RNA is annotated upstream from the nuclease. The HEARO RNA design is shown as inset.

C) Active HEARO RNA designs from cleavage activity assays with active nucleases characterized here.

D) Secondary structure of reference HEARO RNA Ama-1-1 reported by Weinberg et al [3].

E) Secondary structure of the Omega RNAs from two active IscB nucleases reported by Altae-Tran, Kannan et al [2].

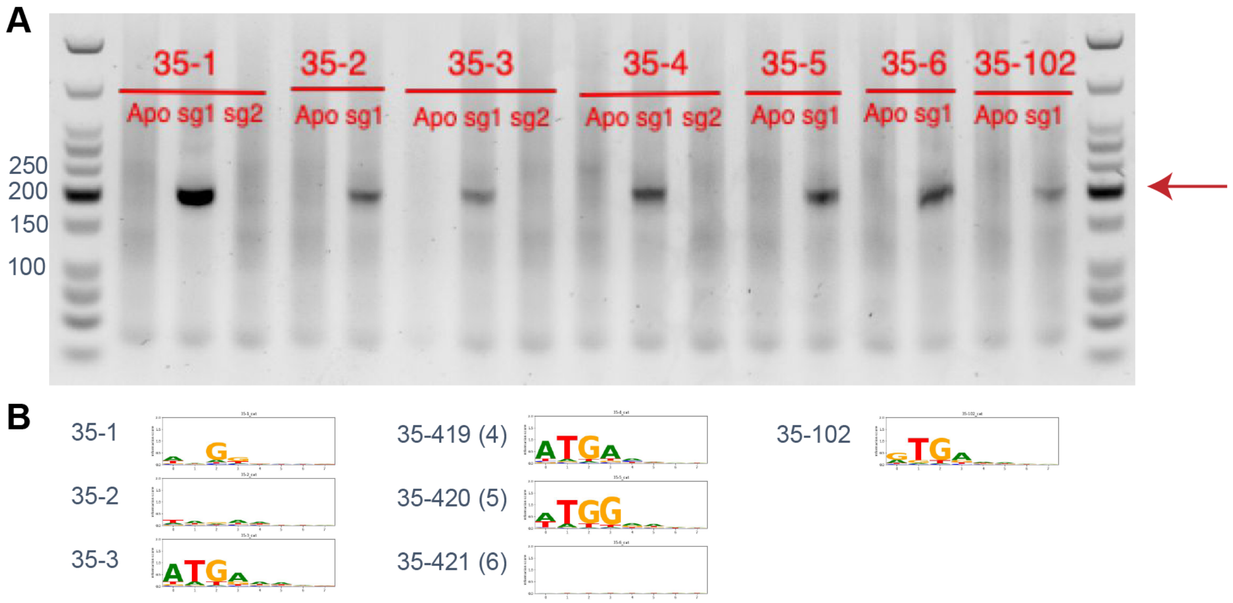

Supplementary Figure 17. HEARO effectors are RNA-guided dsDNA nucleases.

A) Effectors with (sg) and without (Apo) HEARO RNAs were assayed in in-vitro transcription/translation reactions incubated with a PAM library (dsDNA target). Cleavage products were amplified via PCR, where successful RNA guided cleavage by the nuclease produced bands at an expected size (200 bp, marked by red arrow). Sg1 and sg2 are alternate sgRNA designs, where sg2 is a shorter design (inactive). Experiments were repeated twice.

B) Target-adjacent motif (TAM) determined from sequencing of cleavage products from the gel in (A).

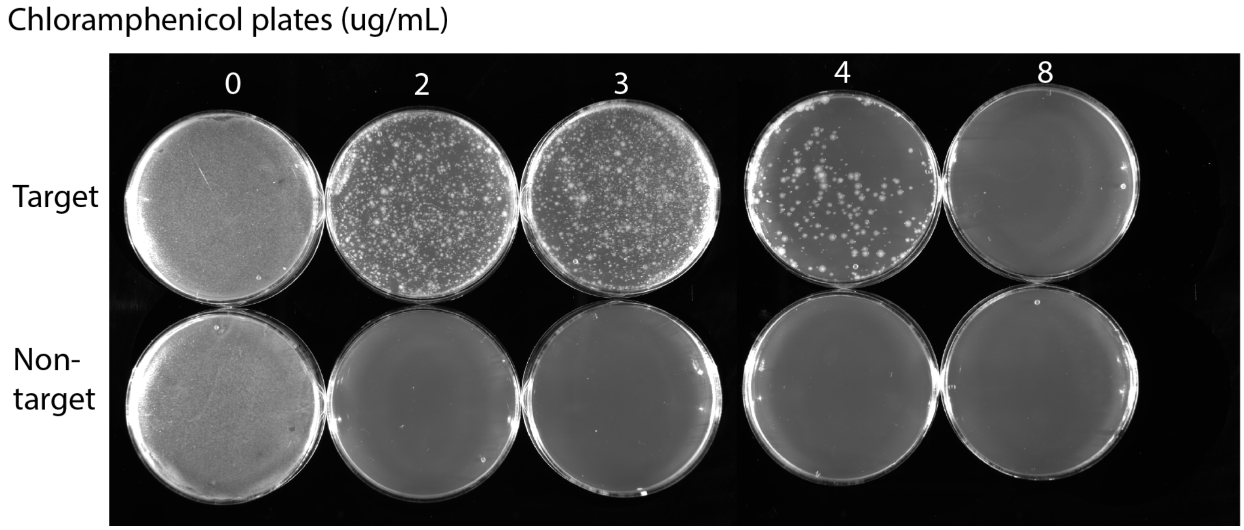

Supplementary Figure 18. *E. coli* survival assay. Original plate images for one of the ABE-MG35-1 replicates. *E. coli* was transformed with a plasmid containing the ABE-MG35-1, a non-functional chloramphenicol acetyltransferase (CAT) gene, and a sgRNA that either targets the CAT gene (target spacer) or not (non-target spacer). *E. coli* survival under chloramphenicol selection is dependent on the ABE-MG35-1 base editing the non-functional CAT gene to its wild-type sequence. Transformed *E. coli* was plated on plates containing chloramphenicol concentrations of 0, 2, 3, 4, and 8 ug/mL. Plates also contain 100 ug/mL Carbecillin and .1mM IPTG. Experiments were performed in duplicate.

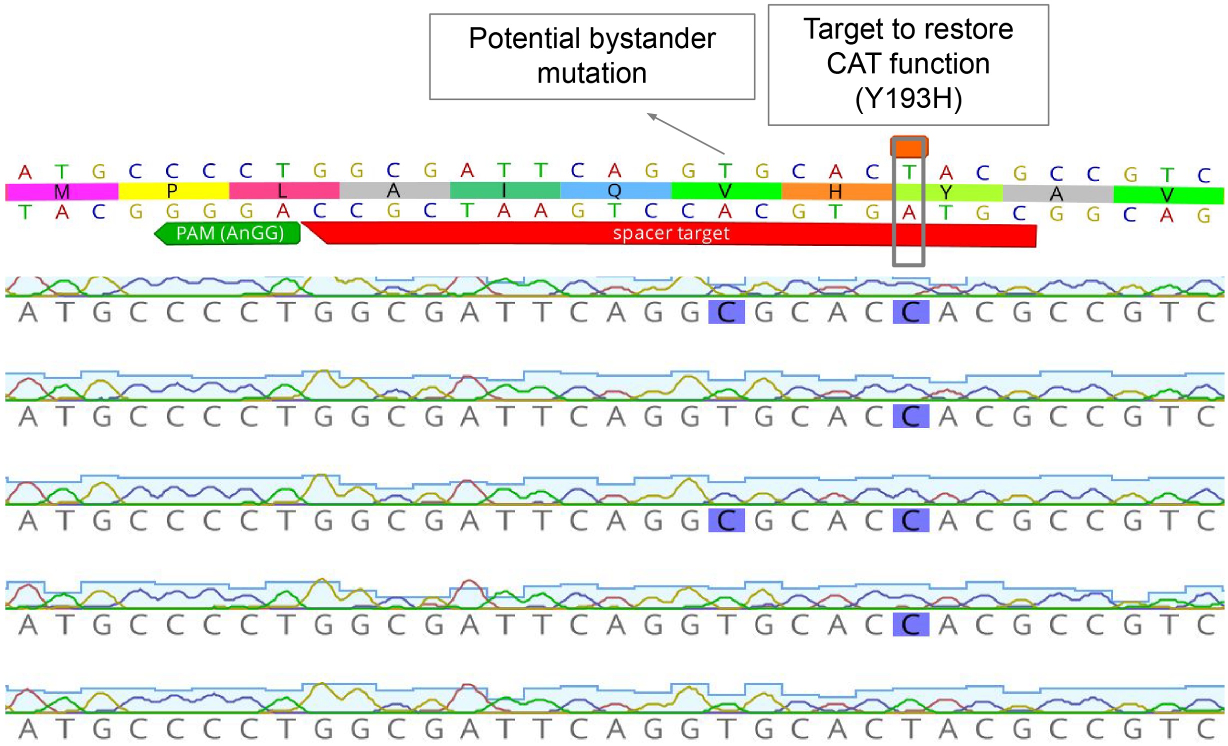

Supplementary Figure 19. ABE-MG35-1 *E. coli* survival assay sequencing results. Colonies grown on plates containing chloramphenicol concentrations of 0, 2, 3, and 4 ug/mL were sequenced to assess reversion of the CAT gene. 26 surviving colonies were picked from plates under chloramphenicol selection from the first experimental replicate and subject to Sanger sequencing. Image of sequencing results of four of five selected colonies show a mutation from A to G on the negative strand, restoring CAT function from Y193 back to H on the positive strand (boxed nucleotides). A bystander base edit was observed in two of the five sequenced colonies.

Supplementary References

1. Burstein, D. *et al.* New CRISPR-Cas systems from uncultivated microbes. *Nature* 542, 237–241 (2017).

2. Altae-Tran, H. *et al.* The widespread IS200/605 transposon family encodes diverse programmable RNA-guided endonucleases. *Science* 374, 57–65 (2021).

3. Weinberg, Z., Perreault, J., Meyer, M. M. & Breaker, R. R. Exceptional structured noncoding RNAs revealed by bacterial metagenome analysis. *Nature* 462, 656–659 (2009).

4. Nishimasu, H. *et al.* Crystal Structure of Cas9 in Complex with Guide RNA and Target DNA. *Cell* 156, 935–949 (2014).

5. Schuler, G., Hu, C. & Ke, A. Structural basis for RNA-guided DNA cleavage by IscB-ωRNA and mechanistic comparison with Cas9. *Sci New York N Y* 376, 1476–1481 (2022).

6. Carugo, O. Amino acid composition and protein dimension. *Protein Sci Publ Protein Soc* 17, 2187–91 (2008).

7. Nowak, C. M., Lawson, S., Zerez, M. & Bleris, L. Guide RNA engineering for versatile Cas9 functionality. *Nucleic Acids Res* 44, 9555–9564 (2016).

8. Kim, E. *et al.* In vivo genome editing with a small Cas9 orthologue derived from Campylobacter jejuni. *Nat Commun* 8, 14500 (2017).

9. Goltsman, D. S. A. *et al.* Novel Type V-A CRISPR Effectors Are Active Nucleases with Expanded Targeting Capabilities. *Crispr J* 3, 454–461 (2020).
