# Supplementary material for: Compact Cas9d and HEARO enzymes for genome editing discovered from uncultivated microbes
Source: Nat Commun. 2022 Dec 15;13:7602. doi: 10.1038/s41467-022-35257-7 (PMC9755519; doi:10.1038/s41467-022-35257-7)
Supplement: Supplementary file 3 — Description of Additional Supplementary Files [file 41467_2022_35257_MOESM3_ESM.pdf]

Title: Supplementary Data 1.
Description: Protein sequences and taxonomic classification for SMART effectors recovered in this work. Metagenomic sequencing accession numbers are included (Excel).

Title: Supplementary Data 2.
Description: Amino acid composition of SMART nucleases (Excel).

Title: Supplementary Data 3.
Description: Active SMART system information (Excel).

Title: Supplementary Data 4.
Description: Genome binning information for active SMART systems (Excel).
